# Spiroleiferthione A and Oleiferthione A: Two Unusual Isothiocyanate-Derived Thioketone Alkaloids from *Moringa oleifera* Lam. Seeds

**DOI:** 10.3390/ph16030452

**Published:** 2023-03-16

**Authors:** Yueping Jiang, Rong Liu, Ling Huang, Qi Huang, Min Liu, Shao Liu, Jing Li

**Affiliations:** 1 Department of Pharmacy, Xiangya Hospital, Central South University, Changsha 410008, China; jiangyueping@csu.edu.cn (Y.J.);; 2 National Clinical Research Center for Geriatric Disorders, Institute for Rational and Safe Medication Practices, Xiangya Hospital, Central South University, Changsha 410008, China; 3 College of Pharmacy, Dali University, Dali 671000, China

**Keywords:** *Moringa oleifera* Lam. seeds, thioketone alkaloids, isolation, structure elucidation

## Abstract

Spiroleiferthione A (**1**), with a 2-thiohydantoin a heterocyclic spiro skeleton, and oleiferthione A (**2**), an imidazole-2-thione derivative, were isolated from the aqueous extract of *Moringa oleifera* Lam. seeds. The unprecedented structures of **1** and **2** were elucidated by extensive spectroscopic data, X-ray diffraction, and gauge-independent atomic orbital (GIAO) NMR calculation, as well as electronic circular dichroism (ECD) calculation. The structures of **1** and **2** were determined to be (5*R*,7*R*,8*S*)-8-hydroxy-3-(4′-hydroxybenzyl)-7-methyl-2-thioxo-6-oxa-1, 3-diazaspiro [4.4] nonan-4-one, and 1-(4′-hydroxybenzyl)-4,5-dimethyl-1,3-dihydro-2*H*-imidazole-2-thione, respectively. Biosynthetic pathways for **1** and **2** have been proposed. Compounds **1** and **2** are considered to have originated from isothiocyanate and then undergone a series of oxidation and cyclization reactions to form **1** and **2**. Compounds **1** and **2** demonstrated weak inhibition rates of NO production, 42.81 ± 1.56% and 33.53 ± 2.34%, respectively, at a concentration of 50 μM. Additionally, Spiroleiferthione A demonstrated moderate inhibitory activity against high glucose-induced human renal mesangial cell proliferation in a dosage-dependent manner. A wider range of biological activities, and the diabetic nephropathy protective activity of Compound **1** in vivo and its mechanism of action, need further investigation after the sufficient enrichment of Compound **1** or total synthesis.

## 1. Introduction

Thiohydantoins, imidazole-2-thione derivatives, and isosteric analogues of hydantoin exhibit numerous biological activities, such as anticancer, anti-inflammatory, immunoregulatory, and antimicrobial properties [1]. To date, no more than 50 naturally occurring thiohydantoin derivatives have been reported in the literature [2,3,4,5,6,7,8,9,10]. They have mainly been discovered in plants of the Brassicaceae (syn. Cruciferae) family, such as *Lepidium meyenii* Walp., *Armoracia rusticana* P.G. Gaertn, and *Pugionium cornutum* (L.) Gaertn [2,3,4,5,6,7,8,9,10]. Only two imidazole-2-thione derivatives derived from ergothioneine have been isolated from a plant extract, although all previously reported ergothioneine-derived natural products are from fungal and animal sources [11]. These derivatives possess biological activities such as antioxidant, hepatoprotective, and epithelial–mesenchymal transition inhibition [12,13,14].

**Figure 1:**
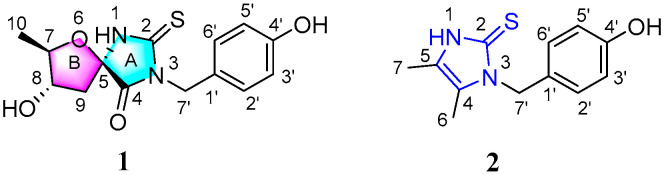
Structures of Compounds **1** and **2**.

*Moringa oleifera* Lam. (Moringaceae), a perennial plant of the Moringaceae family, is widely distributed in Africa and South Asia and is consumed as a folk medicine for the treatment of diabetes, paralysis, helminthiasis, sores, and skin infections, or as food [15,16,17]. *M. oleifera* seeds are an important part of *M. oleifera* and are rich in bioactive compounds and functional ingredients, which makes them a rich source of nutrition with potent therapeutic properties. Pharmacological studies have shown that the extracts of *M. oleifera* seeds, or compounds that were isolated from *M. oleifera* seeds, have a variety of pharmacological activities, namely anti-inflammatory [15,16,18], antiproliferation [15,16,18], hepatoprotective [16,18,19], antiatherosclerotic [16,18,20], antinociceptive [16,18], antidiabetic [18,19,20], antiperoxidative [15,16,18,20], neurodegenerative [18], and cardioprotective properties [15,16,18,20]. Moreover, various chemical constituents have been isolated from the extracts of *M. oleifera* seeds, such as glucosinolates [15,18], flavonoids [15,16,18], carbamates [18], steroids [15,16,18], fatty acids [16,18], phenolics [15,16,18], and polysaccharides [16]. However, the investigation of bioactive compounds and their bioactivities is still inadequate. In order to enrich the structural diversity and in-depth development of *M. oleifera* seeds, it is necessary to continuously determine the active ingredients in *M. oleifera* seeds, especially the sulfur-containing compounds. In our previous studies, the extracts and pyrrole-2-carbaldehydes from the seeds of *M. oleifera* showed strong diabetic-nephropathy-protective and potential neuroprotective activities [21,22]. In order to enrich the scope of active compounds for further pharmacological research, the chemical composition of *M. oleifera* seeds was further investigated, with the identification and isolation of two novel thioketone compounds: Spiroleiferthione A (**1**) and Oleiferthione A (**2**) (Figure 1). Spiroleiferthione A is a rare 2-thiohydantoin with an unprecedented heterocyclic spiro skeleton. Oleiferthione A is a novel, naturally occurring imidazole-2-thione derivative. Notably, Spiroleiferthione A demonstrated moderate inhibitory activity against high glucose-induced human renal mesangial cell (HRMC) proliferation in a dosage-dependent manner. Herein, details of the isolation, structural elucidation, proposed biosynthesis, and bioactivity of the two compounds are described.

## 2. Results

### 2.1. Structural Elucidation

**Figure 2:**
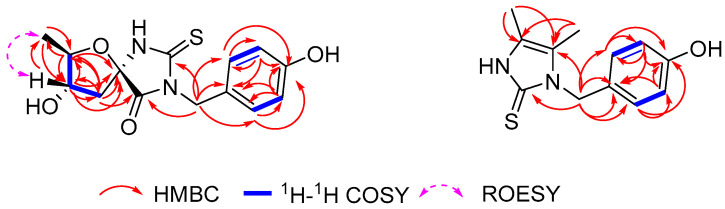
Key 2D NMR correlations of **1** and **2**.

Spiroleiferthione A (**1**) was obtained as a yellowish, amorphous powder with [α${]}_{D}^{25}$+187 (*ⅽ* 0.03, MeOH). Its molecular formula, C_14_H_16_N_2_SO_4_, established by HRESIMS as *m*/*z* 309.0908 [M + H]^+^, combined with NMR spectral data (Table 1), indicates eight degrees of unsaturation. Its IR spectrum shows absorption bands for hydroxy (3415 cm^−1^) and aromatic (1645 and 1563 cm^−1^) functionalities. The ^1^H NMR spectrum of **1** has peaks indicating an aromatic ring AA′BB′ system [*δ*_H_ 7.04 (2H, d, *J* = 8.4 Hz, H-2′, 6′) and *δ*_H_ 6.62 (2H, d, *J* = 8.4 Hz, H-3′, 5′)], one nitrogen-bearing methylene [*δ*_H_ 4.72 (^1^H, d, *J* = 15.0 Hz, H-7′a) and 4.61(^1^H, d, *J* = 15.0 Hz, H-1′b)], one saturated methylene [*δ*_H_ 2.41 (^1^H, dd, *J* = 6.0, 13.2 Hz, H-9a) and 1.95 (^1^H, dd, *J* = 7.2, 13.2 Hz, H-9b)], two nonequivalent methines [*δ*_H_ 3.85 (^1^H, dq, *J* = 6.0, 6.0 Hz, H-7) and 3.83 (^1^H, ddd, *J* = 7.2, 6.0, 6.0 Hz, H-8)], and one methyl [*δ*_H_ 1.09 (3H, d, *J* = 6.0 Hz, H_3_-10)]. The ^13^C NMR and DEPT spectra display signals corresponding to the above proton-bearing units include two additional quaternary carbonyls assigned as carboxylic (*δ*_C_ 172.9, C-4), and thiourea carbonyl (*δ*_C_ 182.6, C-2) groups [3,8,9], and three quaternary carbon resonances that were assigned as two aromatic carbons, including an oxygen-bearing carbon (*δ*_C_ 126.9 and 157.3, respectively) and one nitrogen/oxygen-bearing carbon (*δ*_C_ 90.7) (Table 1). These spectroscopic data, and those of the reported thiohydantoin derivatives (Lee et al., 2019; Peng et al., 2021; Yu et al., 2017) indicate that **1** is a thiohydantoin derivative, as confirmed by 2D NMR data analysis (Figure 2). The HSQC and ^1^H–^1^H COSY spectra of **1** provide the unambiguous assignment of proton and carbon signals in the NMR spectra. In the HMBC spectrum of **1**, correlations from H-2′/6′ to C-1′, C-4′, and C-3′/5′; from H-3′/5′ to C-1′, C-4′, and C-2′/6′; and from H_2_-7′ to C-2, C-4, C-2′/6′, and C-1′ in combination with the chemical shift of H_2_-7′, demonstrate that C-7′ of the 1,4-disubstituted benzyl group is connected to the N-3 atom of the thiohydantoin ring (ring A, Figure 1). Additionally, the thiohydantoin ring (Ring A) is connected to Ring B via C-5, supported by the HMBC correlations of H_2_-9/C-4, C-5, C-7, C-8; H-8/ C-5, C-7, C-9, C-10; H-7/C-5, C-8, C-9, C-10; and H_3_-10/C-7, C-8, in combination with the chemical shift of C-5 (90.7). Hence, the planar structure of **1** was determined to be 8-hydroxy-3-(4′-hydroxybenzyl)-7-methyl-2-thioxo-6-oxa-1, 3-diazaspiro [4.4] nonan-4-one.

**Figure 3:**
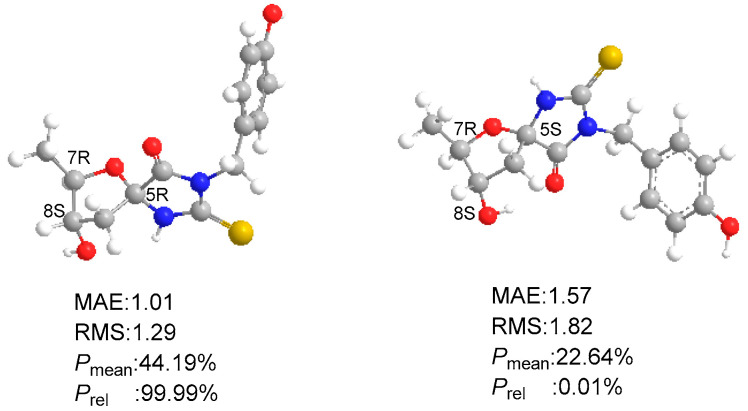
The ^13^C NMR chemical shift calculation results of a pair of C-5 epimers of (5*R*, 7*R*, 8*S*) −**1** (**1a**) and (5*S*, 7*R*, 8*S*) −**1** (**1b**).

The relative configurations of C-7 and C-8 in **1** were determined by the ROESY correlation observed between H-8 and H_3_-10, which suggested that H-8 and CH_3_-10 were on the same side. However, determining the stereochemistry of C-5 was a challenge due to the unprecedented carbon skeleton and the absence of NOEs. To solve this, the ^13^C NMR chemical shifts for the two possible isomers, namely, **1a** [(5*R*,7*R*,8*S*)**1**] and **1b** [(5*S*,7*R*,8*S*) −**1**], were calculated at the ωB97x-D/6-31G*/B3LYP-D3(BJ)/TZVP level [23]. Isomer **1a** yielded a better linear correlation coefficient (R^2^) value (0.9993 vs. 0.9986) (Figure S1), a lower mean absolute error (MAE, 1.01 vs. 1.57), and a lower root mean square (RMS, 1.29 vs. 1.82) than those of Isomer **1b**, suggesting the 5*R*,7*R*,8*S* relative configuration of **1** (Figure 3). Further supporting this conclusion, the experimental and calculated chemical shifts were statistically analyzed using the sorted training set (STS) protocol [23], indicating a striking predominance (99.99% probability) of the (5*R*,7*R*,8*S*) −**1** isomer (Figure 3).

**Figure 4:**
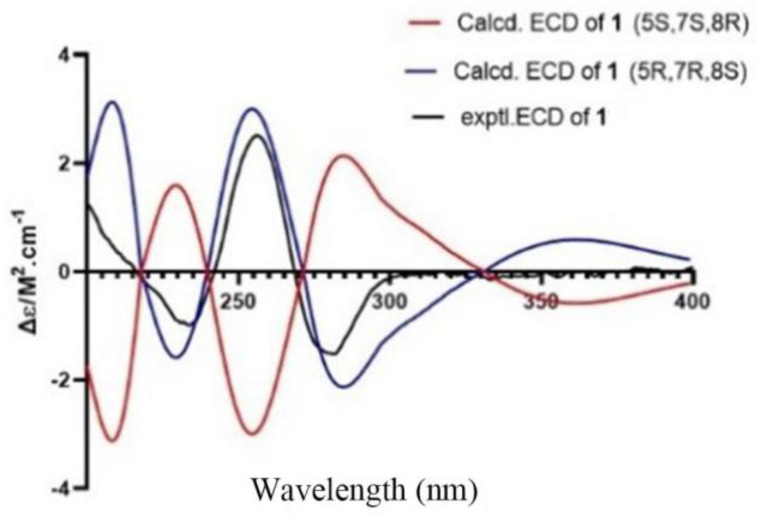
Experimental and calculated ECD spectra of **1**.

ECD calculations were carried out to define the absolute configuration of **1**. As shown in Figure 4, the calculated ECD curve matched well with the experimental data, enabling a confident assignment of the absolute configuration of (5*R*,7*R*,8*S*) −**1**.

Oleiferthione A (**2**), a yellowish, amorphous powder, has the molecular formula C_12_H_14_N_2_OS with seven degrees of unsaturation, as indicated by HRESIMS *m*/*z* 235.0906 [M + H]^+^ (calcd for C_12_H_15_N_2_OS, 235.0905). In the ^1^H NMR spectrum, typical resonance values were observed for two singlet methyls [*δ*_H_ 2.04 (3H, s) and 1.93 (3H, s)], a 1,4-disubstituted benzene ring with four aromatic protons [*δ*_H_ 7.09 (2H, d, *J* = 8.5 Hz) and 6.71 (2H, d, *J* = 8.5 Hz)], and one nitrogen-bearing methylene [*δ*_H_ 5.19 (2H, s)] (Table 2). A *p*-hydroxyphenyl group [*δ*_C_ 128.8 (C-1′), 129.6 (C-2′, 6′), 116.3 (C-3′, 5′), and 158.1 (C-4′)], two methyls [*δ*_C_ 8.8 (C-6) and 9.1 (C-7)], one nitrogen-bearing aliphatic methylene (*δ*_C_ 48.3, C-7′), one nitrogen-bearing olefin [*δ*_C_ 123.6 (C-4) and 121.5 (C-5)], and one thiourea carbonyl, or one sulfhydryl substituted olefinic carbon (*δ*_C_ 159.3, C-2), were identified from the ^13^C NMR, DEPT, and HSQC data (Table 1 and Table 2, Figure S5). These data account for the seven degrees of unsaturation, a pentacyclic ring system satisfying one of these.

From the 2D NMR data, the ^1^H–^1^H COSY correlations of H-2′/H-3′ and H-5′/H-6′ indicated the 1,4-disubstituted benzene ring (Figure 1). The key HMBC correlations from CH_3_-6 to C-4 and C-5; from CH_3_-7 to C-4 and C-5; from H_2_-7′ to C-2, C-4, C-1′, C-2 ′, and C-6′; from H-2′/H-6′ to C-3′/C-5′, C-4′, and C-1′; and from H-3′/H-5′ to C-2′/C-6′, C-1′, and C-4′, in combination with the chemical shift of C-7′, demonstrate that C-7′, in the 1,4-disubstituted benzyl group, is connected to the N-3 atom of the 4,5-dimethyl-1*H*-2-thioimidazole or 4,5-dimethyl-1*H*-imidazole-2-thiol ring. Fortunately, after many attempts, a small crystal of **2** was generated in methanol through the solvent evaporation method. Single-crystal X-ray crystallography using Mo–Kα radiation confirmed the 4,5-dimethyl-1*H*-2-thioimidazole ring in 2 (CCDC: 2168388, Figure 5). Accordingly, the structure of **2**, oleiferthione A, was elucidated as 1-(4′-hydroxybenzyl)-4, 5-dimethyl-1, and 3-dihydro-2*H*-imidazole-2-thione.

### 2.2. Proposed Biosynthetic Pathway of **1** and **2**

**Scheme 1:**
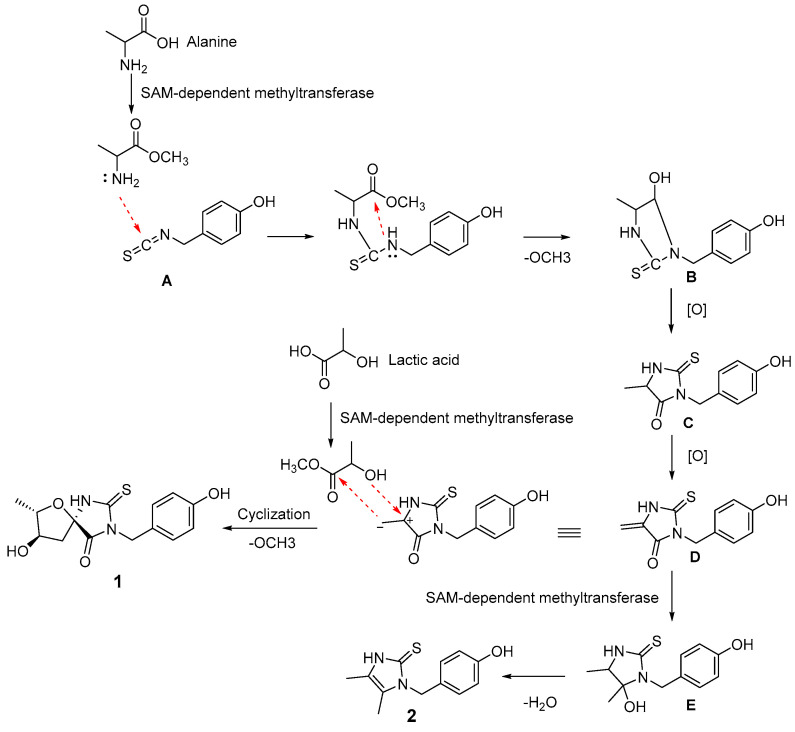
Proposed biosynthetic pathways for **1** and **2**.

Biosynthetic pathways for **1** and **2** are proposed in Scheme 1. The typical isothiocyanate derived from phenylalanine was considered to be the starting unit [24]. The initial head-to-tail cyclization of isothiocyanate (**A**) with methyl alanine would produce **B** with the removal of a methoxy moiety [25]. The oxidation of **B** would yield key intermediate **C**. SAM-dependent methyltransferase would catalyze the conversion of **C** into **E**, which would dehydrate to afford **2**. The oxidation of key intermediate **C** would give **D**. Finally, the cyclization of **D** with methyl lactate would produce **1** in a transformation similar to that producing **B**.

### 2.3. Biological Activity Evaluation of **1** and **2**

**Figure 6:**
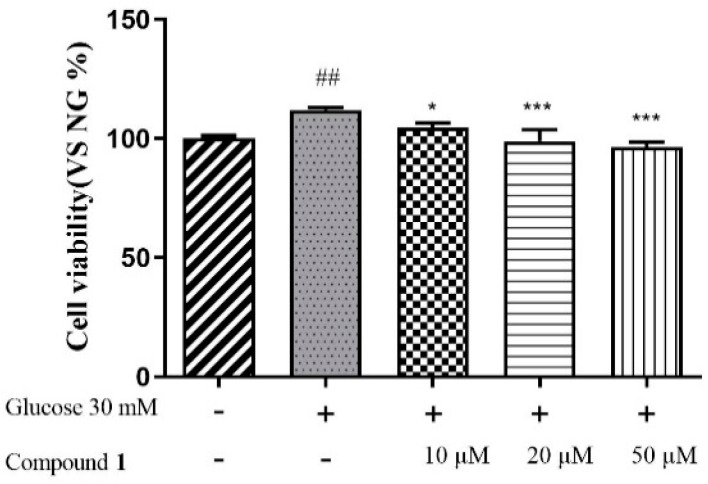
Effects of Compound **1** on the proliferation of glucose-induced HRMCs (* *p* < 0.05, vs. the 30 mM glucose group; *** *p* < 0.001, vs. the 30 mM glucose group; *^##^ p* < 0.01, vs. the control group).

In bioassay experiments, Compounds **1** and **2** were evaluated for their antimicrobial activity and their inhibitory activities against lipopolysaccharide (LPS)-activated nitric oxide (NO) production in RAW 264.7 cells and high glucose-induced human renal mesangial cell (HRMC) proliferation. Compounds **1** and **2** displayed no antimicrobial activity against four bacteria, including *Escherichia coli*, *Staphylococcus aureus* subsp. *Aureus*, *Salmonella enterica* subsp*. Enterica*, and *Pseudomonas aeruginosa* at a concentration of 100 μM. In the antimicrobial assay, the positive control group (Ceftazidime) displayed inhibition rates of 99.853 ± 0.129% and 100.04 ± 0.069% against *E. coli* and *P. aeruginosa*, respectively. Penicillin G sodium salt displayed inhibition rates of 100.232 ± 0.201% and 99.942 ± 0.084% against *S. aureus* and *S. enterica*, respectively. Compounds **1** and **2** showed a weak inhibition of NO production with 42.81 ± 1.56% and 33.53 ± 2.34%, respectively, at a concentration of 50 μM. The positive control (L-NG-monomethylarginine) demonstrated an inhibition of NO production with 59.31 ± 2.19%. However, as shown in Figure 6, Compound **1** showed the moderate inhibition of high glucose-induced HRMC proliferation in a dosage-dependent manner. This result suggests that Compound **1** can potentially exert protective effects against the progression of diabetic nephropathy.

## 3. Discussion

To date, no more than 50 naturally occurring thiohydantoin derivatives have been reported in the literature [2,3,4,5,6,7,8,9,10]. Spiroleiferthione A is a rare 2-thiohydantoin with an unprecedented heterocyclic spiro skeleton that differs from these reported thiohydantoin derivatives. Thus far, only two imidazole-2-thione derivatives derived from ergothioneine have been isolated from a plant extract, although all previously reported ergothioneine-derived natural products are from fungal and animal sources [11]. Oleiferthione A is a novel naturally occurring imidazole-2-thione derivative, which is the third identified plant origin of imidazole-2-thione derivatives. Spiroleiferthione A showed moderate inhibitory activity against high glucose-induced human renal mesangial cell proliferation in a dosage-dependent manner. However, on account of the limitations, the diabetic-nephropathy-protective activity of Compound **1** in vivo and its mechanism of action were not further investigated. Although it has been reported that natural imidazole-2-thione derivatives possess biological activities, such as antioxidant, hepatoprotective, and epithelial–mesenchymal transition inhibition properties [12,13,14], and natural 2-thiohydantoin derivatives showed anti-neuroinflammatory [3,4,8], antibacterial [9], anticancer [8,26], hypolipidemic [2], anticarcinogenic [2], antimutagenic [2], and antithyroidal [2] activities, a wider range of biological effects was not further evaluated due to the limitations of the two compounds. Although various bioactivities of 2-thiohydantoin derivatives have been reported, the antidiabetic nephropathy activity was investigated in this study for the first time. In order to comprehensively investigate its bioactivities and mechanisms of action, the total synthesis of 2-thiohydantoin derivatives may be an effective approach to produce sufficient yields.

## 4. Materials and Methods

### 4.1. General Experimental Procedures

Optical rotations were measured using an INESA SGW-3 automatic polarimeter (Shanghai INESA Physico Optiacal Instrument, Shanghai, China). UV data were recorded using a Cary 300 spectrometer (Agilent Technologies, Santa Clara, CA, USA). CD data were measured using a JASCO J-815 CD spectrometer (JASCO, Tokyo, Japan). IR data were recorded using a Nicolet IS50 FT-IR spectrometer (Thermo Fisher Scientific, Waltham, MA, USA). NMR spectra were obtained at 500 MHz or 600 MHz for ^1^H, and 125 MHz or 150 MHz for ^13^C on Bruker 500 MHz or 600 MHz spectrometers (Bruker, Billerica, MA, USA) in CD_3_OD or DMSO-*d*_6_, respectively, with the tetramethyl silane (TMS) peak used as a reference. HR-ESIMS data were measured using an Agilent Q-TOF 6545 spectrometer (Agilent Technologies). Column chromatography (CC) was performed using silica gel (200−300 mesh, Qingdao Marine Chemical, Qingdao, China), MCI gel (CHP20P) (Mitsubishi Chemical, Tokyo, Japan), and macroporous adsorbent resin (HPD-300) (Ainuo chemical technology Co., LTD, Zhengzhou, China). HPLC separation was performed using a system consisting of an Agilent 1260, an Agilent 1260 pump, and an Agilent 1260 wavelength absorbance detector with an Agilent (250 × 9.4 mm) semipreparative column packed with C_18_ (5 μm) (Agilent Technologies). TLC separations were carried out on precoated silica gel GF254 plates (Qingdao Marine Chemical). Spots were visualized under UV light (254 or 356 nm), or by spraying with 10% H_2_SO_4_ in 90% EtOH followed by heating.

### 4.2. Plant Material

*M. oleifera* (Moringaceae) seeds were collected in September 2019 from Kunming city, Yunnan Province, China (Latitude: 24° 88′ N; Longitude:102° 88′ E). The seeds were dried at 50 °C in an oven. Plant identity was verified by Professor Shao Liu (Xiangya Hospital, Central South University, Changsha 410008, China). A voucher specimen (no. 2019001) was deposited at the Department of Pharmacy, Xiangya Hospital, Central South University, Changsha 410008, China.

### 4.3. Extraction and Isolation

Dry ground *M. oleifera* seeds (10 kg) were extracted with water using ultrasonic apparatus (solvent ratio: 1:8 (g seeds: mL H_2_O), 3 × 1 h). The aqueous extracts were evaporated under reduced pressure and lyophilized to yield a dark brown residue (2.65 kg). The residue was dissolved in H_2_O (20 L), loaded on a macroporous adsorbent resin (HPD-300) column (10 × 80 cm), and successively eluted with H_2_O (80 L), EtOH-H_2_O (1:1) (80 L) and EtOH-H_2_O (9:1) (50 L) to yield three corresponding fractions: A, B, and C. After removing the solvent under reduced pressure, Fraction A (1.5 kg) was separated by CC over MCI gel CHP 20P (5 L), with successive elution using H_2_O (20 L), EtOH-H_2_O (1:1) (20 L), and EtOH-H_2_O (9:1) (20 L) to yield fractions A1−A3. Fraction A2 (104 g) was subjected to CC over RP silica gel (1 L), with successive elution using EtOH-H_2_O (3:7) (5L), EtOH-H_2_O (1:1) (5 L), and MeOH (5 L) to give fractions A2-1−A2-3. Fraction A2-2 (12 g) was subjected to CC over silica gel, with elution by a gradient of increasing MeOH concentration (0−100%) in CH_2_Cl_2_ to yield fractions A2-2-1−A2-2-12 based on TLC analysis. A2-2-7 (~100 mg) was purified by RP HPLC (C_18_ column, 2.0 mL/min), using 23% MeCN in H_2_O containing 1 ‰ formic acid (*v*/*v*) as the mobile phase to yield **1** (1.5 mg, *t*_R_ = 36.5 min). A2-2-6 (~150 mg) was purified by RP HPLC (C_18_ column, 2.0 mL/min), using 20% MeCN in H_2_O containing 1‰ formic acid (*v*/*v*) as the mobile phase for the former to yield **2** (2.0 mg, *t*_R_ = 27.5 min).

### 4.4. Physicochemical Properties of Compounds **1** and **2**

Spiroleiferthione A (**1**): yellowish, amorphous powder; UV (MeOH) λ_max_ 280 nm; IR (KBr) ν_max_ 3415, 2930, 2856, 1645, 1563, 1439, 1384, 1259, 1152, 1101, 800, 649, 600, 466 cm^−1^; ^1^H NMR (CD_3_OD, 500 MHz and DMSO-*d*_6_, 600MHz) data, see Table 1; ^13^C NMR (CD_3_OD, 125 MHz and DMSO-*d*_6_, 150MHz) data, see Table 1; (+)-HRESIMS *m*/*z* 309.0905 [M + H]^+^ (calcd for C_14_H_16_N_2_O_4_S, 309.0904).

Oleiferthione A (**2**): yellowish, amorphous powder; UV (MeOH) λ_max_ 270 nm; IR (KBr) ν_max_ 3448, 2925, 2850, 1633, 1514, 1456, 1399, 1242, 1168, 1114, 787, 665, 544 cm^−1^; ^1^H NMR (CD_3_OD, 500 MHz) data, see Table 1; ^13^C NMR (CD_3_OD, 125MHz) data, see Table 1; (+)-HRESIMS *m*/*z* 235.0901 [M + H]^+^ (calcd for C_12_H_14_N_2_OS, 235.0900).

Crystallographic and structure refinement for Compound **2**: C_12_H_14_N_2_OS, M=235.02, *a* = 12.2627(6) Å, *b* = 6.2696(3) Å, *c* = 16.1773(7) Å, α = 90°, β = 106.647°(3), γ = 90°, V = 1191.62(10) Å^3^, T = 296.15 K, Space Group Pn, Z = 4, μ(MoKα) = 0.253/mm^−1^, 6212 reflections collected, 3840 independent reflections (R*int* = 0.0282). The final R_1_ value was 0.0535 (*I* > 2σ(*I*). The final *w*R (F^2^) values were 0.1217 (*I* > 2σ(*I*). The final R_1_ value was 0.0993 (all data). The final *w*R (F^2^) value was 0.1502 (all data). The goodness of fit value on F^2^ was 1.018.

### 4.5. Antimicrobial Assay

The microorganisms used in antibacterial assays were obtained from the China General Microbiological Culture Collection Center (CGMCC) (*Escherichia coli* ATCC25922, *Staphylococcus aureus* subsp. *Aureus* ATCC29213, *Salmonella enterica* subsp. *enterica* ATCC14028, and *Pseudomonas aeruginosa* ATCC27853. Lysogeny broth (LB, Huankai Microbial, Guangzhou, China) culture media were used for culturing bacteria, while Mueller–Hinton broth (MHB, Huankai Microbial, Guangzhou, China) was used to determine the minimum inhibitory concentration. The inocula of bacteria was prepared from 24 h-old agar cultures and incubated to logarithmic phase. Ceftazidime (Yuanye biotechnology Co., LTD, Shanghai, China) was used as a positive reference substance. We measured the minimum inhibitory concentration (MIC) of Compounds **1** and **2** using the broth microdilution method in 96-well microtiter plates, as described in the literature [27]. The MIC was defined as the lowest concentration without any colony growth after incubating the fungus at 28 °C for 16 h. All tests were performed in triplicates.

### 4.6. Anti-Inflammatory Assay

Murine RAW 264.7 cells were purchased from the Shanghai cell bank of the Chinese Academy of Sciences (Shanghai, China). These cells were incubated in RPMI1640 medium plus 10% FBS, 100 U/mL penicillin, and 100 μg/mL streptomycin sulfate in a humidified incubator with 5% CO_2_. The cells were treated with LPS (1 µL/mL) and the test compounds for 48 h. L-NMMA was used as a positive control. Accumulated nitrite in the culture supernatants, an indicator of NO synthase activity, was measured by the Griess reaction [28]. Cell viability was examined by MTS assay (Beyotime Inst. Biotech. Shanghai, China) according to the manufacturer’s instructions. The absorbance was examined at 570 nm using a Spectra Max microplate reader (Molecular Devices, LLC, Sunnyvale, CA, USA). All tests were performed in triplicates. The results were expressed as a percentage of the response of the related LPS-treated groups that were designated as 100%.

### 4.7. Inhibitory Assay of High Glucose-Induced HRMC Proliferation

HRMCs were cultured in DMEM (Sigma, St. Louis, MO, USA) and supplemented with 10% fetal bovine serum (FBS, Gibco, CA, USA) and 1% double antibiotic (penicillin–streptomycin, Beyotime Biotechnology Co., Ltd., Shanghai, China) at 37 °C in a humidified environment containing 5% CO_2_. Next, 20 μL of L-DMEM was added to the blank and control wells, and 10 μL each of L-DMEM and 250 mM glucose (0.26 g of D-(+)-glucose dissolved in 8 mL of phosphate-buffered saline (PBS) and filtered using a 0.22 μm membrane) was added to the other wells to a final glucose concentration of 30 mM, which were then incubated for 24 h [22]. Different concentrations of Compounds **1** and **2** (10, 20, and 50 μM) were added to the designated wells, and the Cell Counting Kit-8 (CCK8, Dojindo, Kyushu Island, Japan) assay was used to detect optical density after 24 h of incubation. We used L-DMEM containing only HRMCs and no intervention drugs as a control group.

### 4.8. Statistical Analysis

One-way ANOVAs and LSD t-tests were used to analyze the groups of samples using SPSS 19.0. The data are expressed as the mean ± SD (standard deviation). All the data shown are representative of at least three independent experiments. *p* < 0.05 was considered to be statistically significant.

### 4.9. Computational Section

Crest software was used to search the conformers of (5*R*,7*R*,8*S*)-**1** and (5*S*, 7*R*, 8*S*)-**1** on the GFNFF level of theory [29,30], following the optimization of GFN2-XTB level with a 4 kcal/mol energy window to remove high-energy conformers [31]. Optimization and frequency calculations of each conformer were performed on B3LYP-D3(BJ)/TZVP (IEFPCM, CH_3_OH) level of theory. DFT GIAO ^13^C NMR calculations were performed on the ωB97xD/6-31G * (IEFPCM, CH_3_OH) level, and data processing followed the reported STS protocol [23]. The calculated shielding tensors of conformers were Boltzmann averages based on the Gibbs free energy. Theoretical ECD was calculated by time-dependent density functional theory (TDDFT) at the mPW1PW91/6-311g(d) level with the IEF–PCM solvent model (MeOH). SpecDis v1.71 was used to simulate the ECD for (5R,7R,8S)-**1** curve with Gaussian band shape at 0.28 eV. The calculated ECD curve of each conformer was Boltzmann-averaged based on its Gibbs free energy. All the DFT calculations were performed using the Gaussian 16 software package.

## 5. Conclusions

Two isothiocyanate-derived thioketone alkaloids, Spiroleiferthione A and Oleiferthione A, were isolated from the aqueous extract of *M. oleifera* seeds. The novel structures of **1** and **2** were elucidated by extensive spectroscopic data, gauge-independent atomic orbital (GIAO) NMR calculation, ECD, and X-ray diffraction. Spiroleiferthione A is a rare 2-thiohydantoin with an unprecedented heterocyclic spiro skeleton. Oleiferthione A is a novel, naturally occurring imidazole-2-thione derivative. Spiroleiferthione A demonstrated moderate inhibitory activity against high glucose-induced human renal mesangial cell proliferation in a dosage-dependent manner. This study has enriched the structural diversity of *M. oleifera* seeds, especially the isothiocyanate-derived, sulfur-containing compounds. It provides clues for the subsequent study of thioketone derivatives and their activities.

## Figures and Tables

**Figure 5:**
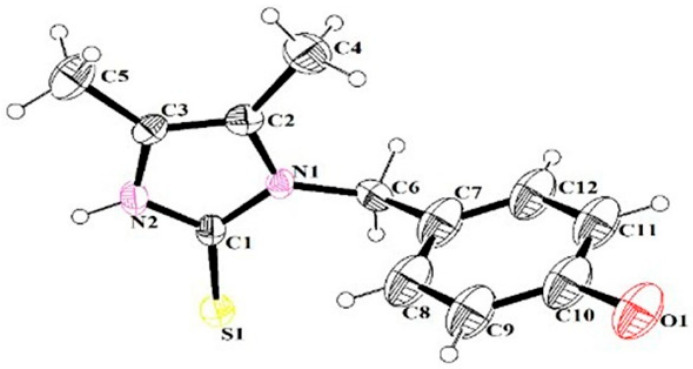
ORTEP diagram of Compound **2**.

**Table 1:** ^1^H and ^13^C NMR data of Compound **1**.

no.	*δ*_H_ (*J* in Hz) ^a^	δ_C_^a^	*δ*_H_ (*J* in Hz) ^b^	*δ*_C_^b^
2		184.3		182.6
4		174.3		172.9
5		92.5		90.7
7	4.13 m	85.0	3.85 dq (6.0, 6.0)	82.1
8	4.14 m	76.8	3.83 ddd (7.2, 6.0, 6.0)	75.4
9a	2.53 dd (13.5, 5.5)	42.0	2.41 dd (13.2, 6.0)	41.2
9b	2.06 dd (13.5, 5.0)		1.95 dd (13.2, 7.2)	
10	1.22 d (6.0)	19.2	1.09 d (6.0)	18.8
1′		128.5		126.9
2′	7.22 d (8.5)	130.9	7.04 d (8.4)	129.7
3′	6.69 d (8.5)	116.1	6.62 d (8.4)	115.6
4′		158.1		157.3
5′	6.69 d (8.5)	116.1	6.62 d (8.4)	115.6
6′	7.22 d (8.5)	130.9	7.04 d (8.4)	129.7
7′a	4.86 overlapped	44.5	4.72 d (15.0)	43.3
7′b			4.61 d (15.0)	

^a^ 500 MHz for ^1^H NMR, 125 MHz for ^13^C NMR, in CD_3_OD; ^b^ 600 MHz for ^1^H NMR, 150 MHz for ^13^C NMR, in DMSO-*d*_6_. Assignments were determined through a combination of 1D and 2D NMR experiments.

**Table 2:** ^1^H and ^13^C NMR data of compound **2** in CD_3_OD.

No.	δ_H_ (J in Hz) ^a^	δ_C_ ^a^
2		159.3
4		123.6
5		121.5
6	2.04 s	8.8
7	1.93 s	9.1
1′		128.8
2′	7.09 d (8.5)	129.6
3′	6.71 d (8.5)	116.3
4′		158.1
5′	6.71 d (8.5)	116.3
6′	7.09 d (8.5)	129.6
7′	5.19 s	48.3

^a^ 500 MHz for ^1^H NMR, 125 MHz for ^13^C NMR, in CD_3_OD.

## Data Availability

Data is contained within the article and Supplementary Materials.

## Supplementary Materials

The following supporting information can be downloaded at: https://www.mdpi.com/article/10.3390/ph16030452/s1, Figure S1: The linear correlation plots of predicted versus experimental ^13^C NMR chemical shifts. (Left (5R, 7R, 8S)-**1**, Right (5S, 7R, 8S)-**1**). Figures S2–S8: NMR, IR, UV, and MS spectra; Table S1: Geometry data of conformers of isomer **1a** and isomer **1b**; Figure S2. (+)-HRESIMS spectrum of compound **1**; Figure S3. (+)-HRESIMS spectrum of compound **1**; Figure S4. The UV spectrum of compound **1** in MeOH; Figure S5. The IR spectrum of compound **1**; Figure S6. The ^1^H NMR spectrum of compound **1** in MeOH-d4 (500 MHz); Figure S7. The ^13^C NMR spectrum of compound **1** in MeOH-d4 (125 MHz); Figure S8. The ^1^H-^1^H COSY spectrum of compound **1** in MeOH-d4 (500 MHz); Figure S9. The HSQC spectrum of compound **1** in MeOH-d4 (500 MHz for ^1^H); Figure S10. The HMBC spectrum of compound **1** in MeOH-d4 (500 MHz for ^1^H); Figure S11. The ROESY spectrum and enlarged ROESY spectrum of compound **1** in MeOH-d4 (500 MHz); Figure S12. The ^1^H NMR spectrum of compound **1** in DMSO-d6 (600 MHz); Figure S13. The ^13^C NMR spectrum of compound **1** in DMSO-d6 (150 MHz); Figure S14. The DEPT spectrum of compound **1** in DMSO-d6 (150 MHz); Figure S15. The ^1^H-^1^H COSY spectrum of compound **1** in DMSO-d6 (600 MHz); Figure S16. The HSQC spectrum of compound **1** in DMSO-d6 (600 MHz for ^1^H); Figure S17. The HMBC spectrum of compound **1** in DMSO-d6 (600 MHz for ^1^H); Figure S18. The NOESY spectrum and enlarged NOESY spectrum of compound **1** in DMSO-d6 (600 MHz); Figure S19. (+)-HRESIMS spectrum of compound 2 (page 1); Figure S20. (+)-HRESIMS spectrum of compound **2** (page 2); Figure S21. The UV spectrum of compound **2** in MeOH; Figure S22. The IR spectrum of compound **2**; Figure S23. The ^1^H NMR spectrum of compound **2** in MeOH-d4 (500 MHz); Figure S24. The ^13^C NMR spectrum of compound **2** in MeOH-d4 (125 MHz); Figure S25. The DEPT spectrum of compound **2** in MeOH-d4 (125 MHz); Figure S26. The ^1^H-^1^H COSY spectrum of compound **2** in MeOH-d4 (500 MHz); Figure S27. The HSQC spectrum of compound **2** in MeOH-d4 (500 MHz for ^1^H); Figure S28. The HMBC spectrum of compound **2** in MeOH-d4 (500 MHz for ^1^H); Table S1. Geometry data of conformers of isomer **1a** and isomer **1b**.
