# Supplementary material for: Spiroleiferthione A and Oleiferthione A: Two Unusual Isothiocyanate-Derived Thioketone Alkaloids from Moringa oleifera Lam. Seeds
Source: Pharmaceuticals (Basel). 2023 Mar 16;16(3):452. doi: 10.3390/ph16030452 (PMC10054748; doi:10.3390/ph16030452)
Supplement: Supplementary file 1 [file pharmaceuticals-16-00452-s001.zip › pharmaceuticals-2235580-supplementary.pdf]

Spiroleiferthione A and Oleiferthione A:Two Isothiocyanate-derived Thioketone Alkaloids from *Moringa oleifera* Lam. Seeds

Yueping Jiang,[a,b] Rong Liu,[a,b] Lin Huang,[c] Qi Huang,[a,b] Min Liu,[a,b] Shao Liu,[*, a,b] and Jing Li[*, a,b]

[a] Department of Pharmacy, Xiangya Hospital, Central South University, Changsha, 410008, China

[b] Institute for Rational and Safe Medication Practices, National Clinical Research Center for Geriatric Disorders, Xiangya Hospital, Central South University, Changsha,410008, China

[c] College of Pharmacy, Dali University, Dali, 671000, China

Corresponding Author

*E-mail: liushao999@csu.edu.cn (S. Liu)

*E-mail: lijingliyun@163.com (J. Li)

Supporting Information

Table of Contents

No.	Contents	Page
1	**Figure S1.** The linear correlation plots of predicted versus experimental ^{13}C NMR chemical shifts. (Left (5R, 7R, 8S)-**1**, Right (5S, 7R, 8S)-**1**).	S3
2	**Figure S2**. (+)-HRESIMS spectrum of compound **1** (page 1).	S4
3	**Figure S3**. (+)-HRESIMS spectrum of compound **1** (page 2).	S5
4	**Figure S4**. The UV spectrum of compound **1** in MeOH.	S6
5	**Figure S5**. The IR spectrum of compound **1**.	S6
6	**Figure S6**. The ^{1}H NMR spectrum of compound **1** in MeOH-d_4 (500 MHz).	S7
7	**Figure S7**. The ^{13}C NMR spectrum of compound **1** in MeOH-d_4 (125 MHz).	S7
8	**Figure S8**. The ^{1}H-^{1}H COSY spectrum of compound **1** in MeOH-d_4 (500 MHz).	S8
9	**Figure S9**. The HSQC spectrum of compound **1** in MeOH-d_4 (500 MHz for ^{1}H).	S8
10	**Figure S10**. The HMBC spectrum of compound **1** in MeOH-d_4 (500 MHz for ^{1}H).	S9
11	**Figure S11**. The ROESY spectrum and enlarged ROESY spectrum of compound **1** in MeOH-d_4 (500 MHz).	S9
12	**Figure S12**. The ^{1}H NMR spectrum of compound **1** in DMSO-d_6 (600 MHz).	S10
13	**Figure S13**. The ^{13}C NMR spectrum of compound **1** in DMSO-d_6 (150 MHz).	S10
14	**Figure S14**. The DEPT spectrum of compound **1** in DMSO-d_6 (150 MHz).	S11
15	**Figure S15**. The ^{1}H-^{1}H COSY spectrum of compound **1** in DMSO-d_6 (600 MHz).	S11
16	**Figure S16**. The HSQC spectrum of compound **1** in DMSO-d_6 (600 MHz for ^{1}H).	S12
17	**Figure S17**. The HMBC spectrum of compound **1** in DMSO-d_6 (600 MHz for ^{1}H).	S12
18	**Figure S18**. The NOESY spectrum and enlarged NOESY spectrum of compound **1** in DMSO-d_6 (600 MHz).	S13
19	**Figure S19**. (+)-HRESIMS spectrum of compound **2** (page 1).	S14
20	**Figure S20**. (+)-HRESIMS spectrum of compound **2** (page 2).	S15
21	**Figure S21**. The UV spectrum of compound **2** in MeOH.	S16
22	**Figure S22**. The IR spectrum of compound **2**.	S16
23	**Figure S23**. The ^{1}H NMR spectrum of compound **2** in MeOH-d_4 (500 MHz).	S17

24	**Figure S24**. The ^{13}C NMR spectrum of compound **2** in MeOH-d_4 (125 MHz).	S17
25	**Figure S25**. The DEPT spectrum of compound **2** in MeOH-d_4 (125 MHz).	S18
26	**Figure S26**. The ^1H-^1H COSY spectrum of compound **2** in MeOH-d_4 (500 MHz).	S18
27	**Figure S27**. The HSQC spectrum of compound **2** in MeOH-d_4 (500 MHz for ^1H).	S19
28	**Figure S28**. The HMBC spectrum of compound **2** in MeOH-d_4 (500 MHz for ^1H).	S19
29	**Table S1**. Geometry data of conformers of isomer **1a** and isomer **1b**.	S20

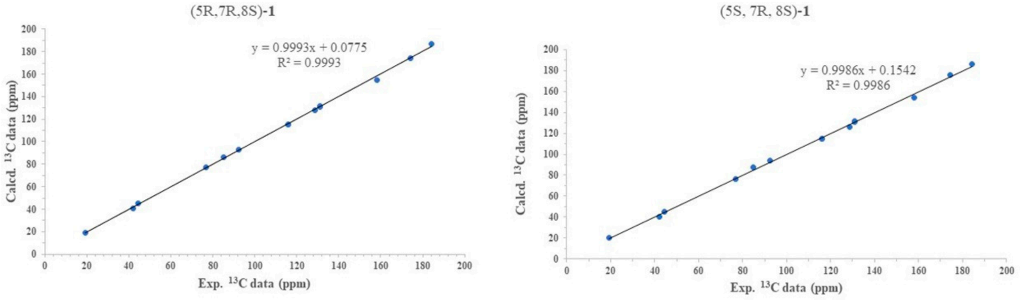

Figure S1. The linear correlation plots of predicted versus experimental ^{13}C NMR chemical shifts. (Left (5R, 7R, 8S)-**1**, Right (5S, 7R, 8S)-**1**).

Qualitative Analysis Report

Chromatograms

Fragmentor Voltage 110 **Collision Energy** 0 **Ionization Mode** ESI

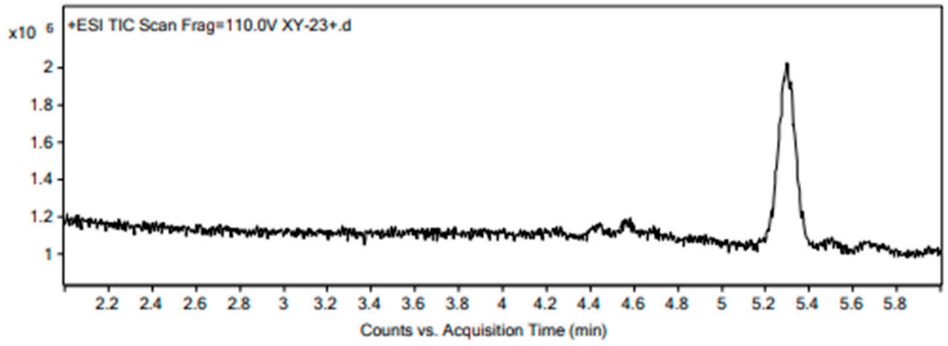

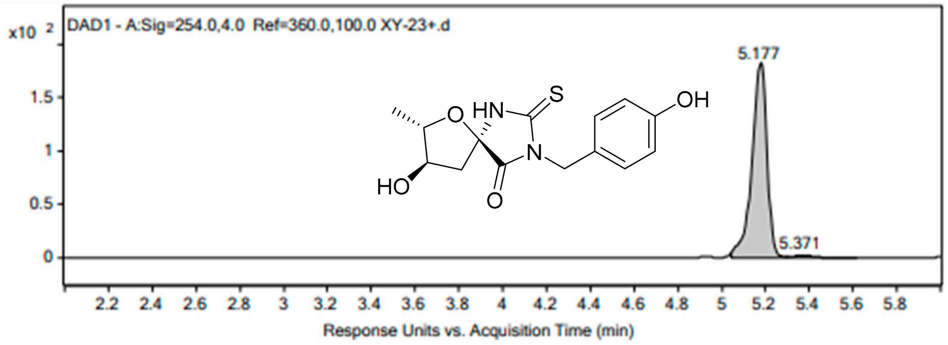

Spectra

Fragmentor Voltage	Collision Energy	Ionization Mode
110	0	ESI

Figure S2. (+)-HRESIMS spectrum of compound **1** (page 1).

Qualitative Analysis Report

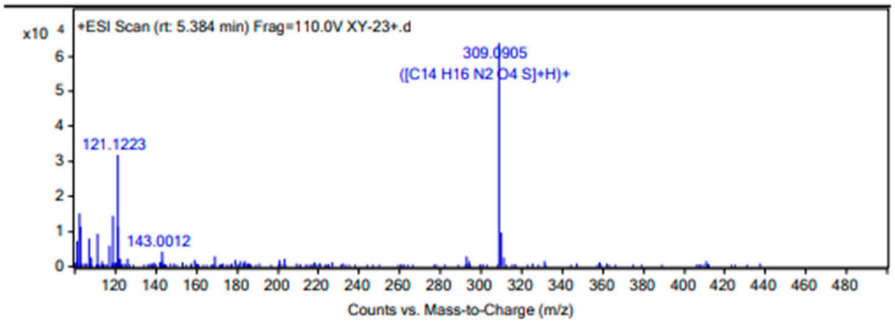

Formula Calculator Element Limits

Element	Min	Max
C	0	60
H	0	120
O	0	10
N	0	5
S	0	5

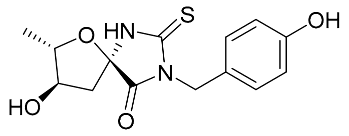

Formula Calculator Results

Formula	Best	Mass	Tgt Mass	Diff (ppm)	Ion Species	Score
C14 H16 N2 O4 S	True	308.0832	308.0831	-0.28	C14 H17 N2 O4 S	97.22
C12 H14 N5 O3 S	False	308.0832	308.0817	-4.89	C12 H15 N5 O3 S	91.05
C8 H14 N5 O8	False	308.0831	308.0842	3.85	C8 H15 N5 O8	87.07
C20 H10 N3 O	False	308.0829	308.0824	-1.69	C20 H11 N3 O	82.1
C6 H20 N4 O6 S2	False	308.0834	308.0824	-3.02	C6 H21 N4 O6 S2	79.47
C9 H18 N5 O3 S2	False	308.0833	308.0851	5.75	C9 H19 N5 O3 S2	78.19
C22 H12 O2	False	308.0829	308.0837	2.84	C22 H13 O2	77.78
C11 H18 N O7 S	False	308.0832	308.0804	-8.94	C11 H19 N O7 S	76.14
C17 H14 N3 O S	False	308.0832	308.0858	8.42	C17 H15 N3 O S	75
C10 H16 N2 O9	False	308.083	308.0856	8.53	C10 H17 N2 O9	74.86

--- End Of Report ---

Figure S3. (+)-HRESIMS spectrum of compound **1** (page 2).

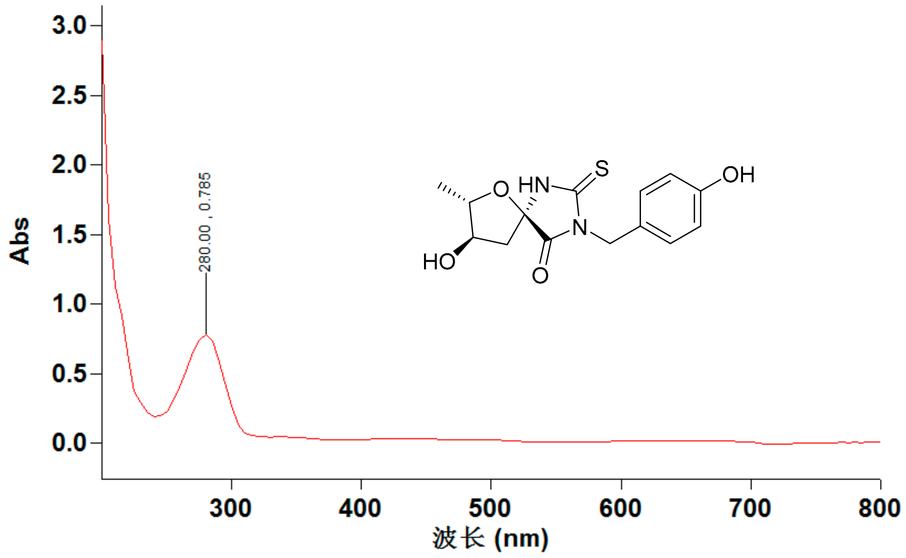

Figure S4. The UV spectrum of compound **1** in MeOH.

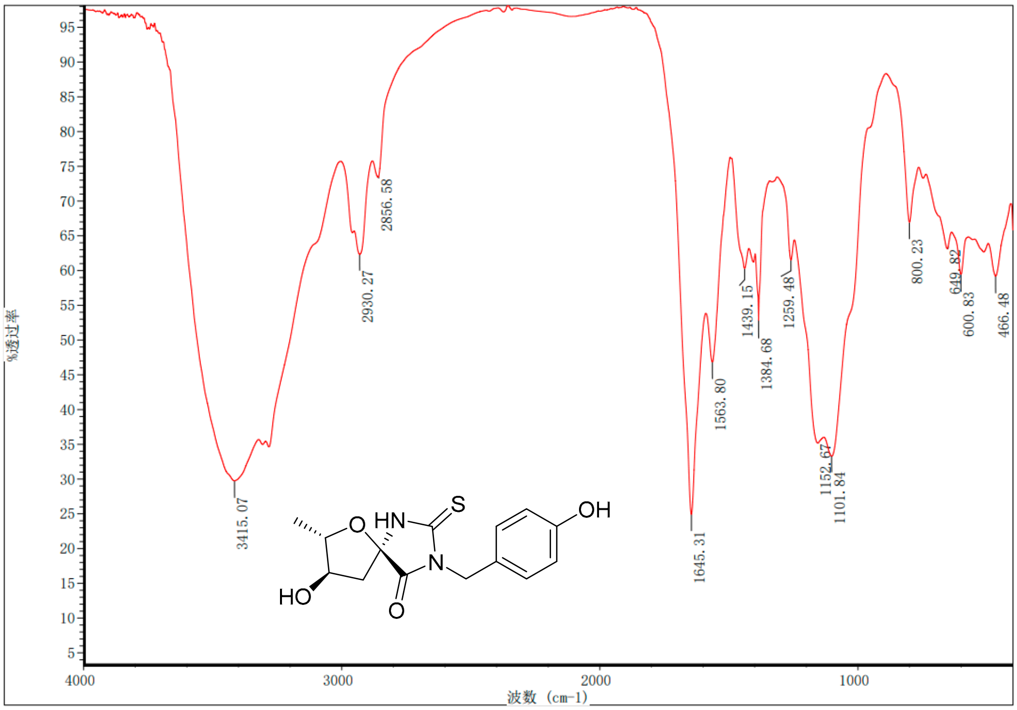

Figure S5. The IR spectrum of compound **1**.

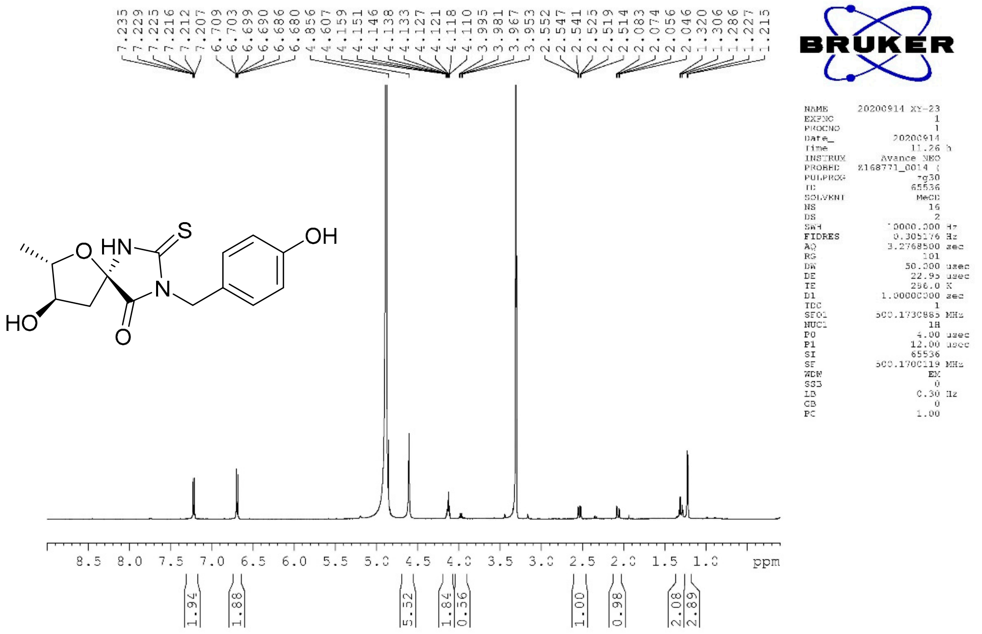

Figure S6. The ^{1}H NMR spectrum of compound **1** in MeOH-d_4 (500 MHz).

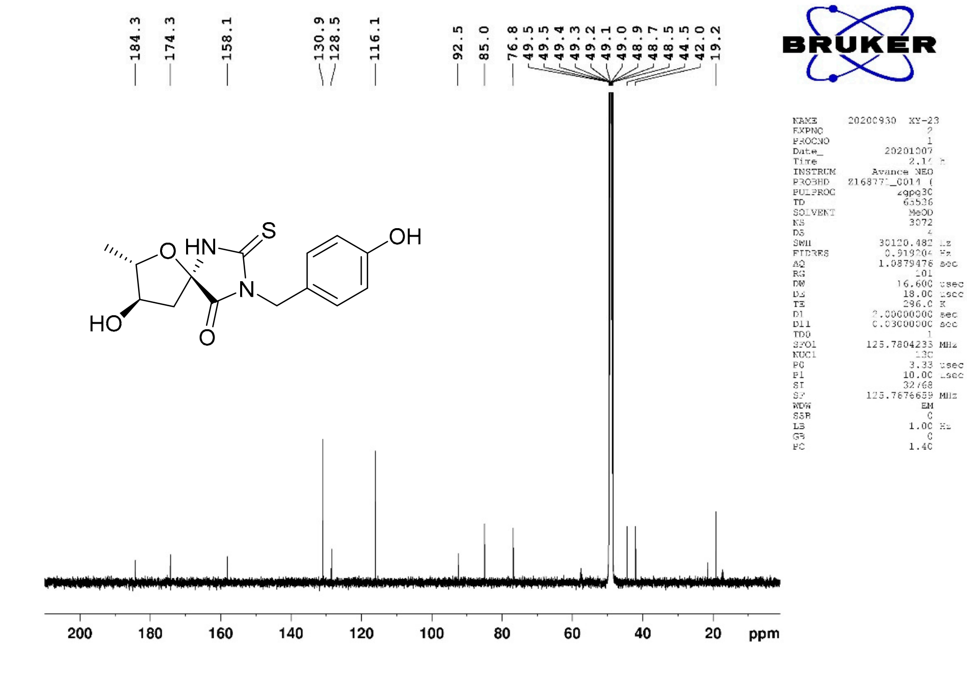

Figure S7. The ^{13}C NMR spectrum of compound **1** in MeOH-d_4 (125 MHz).

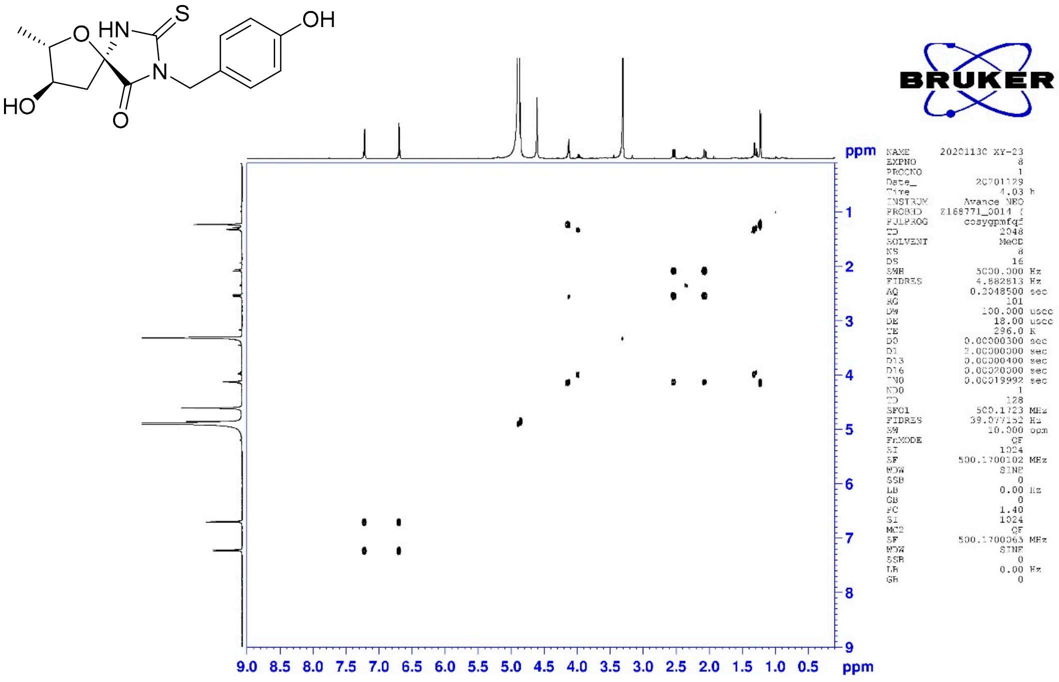

Figure S8. The ^{1}H-^{1}H COSY spectrum of compound **1** in MeOH-d_4 (500 MHz).

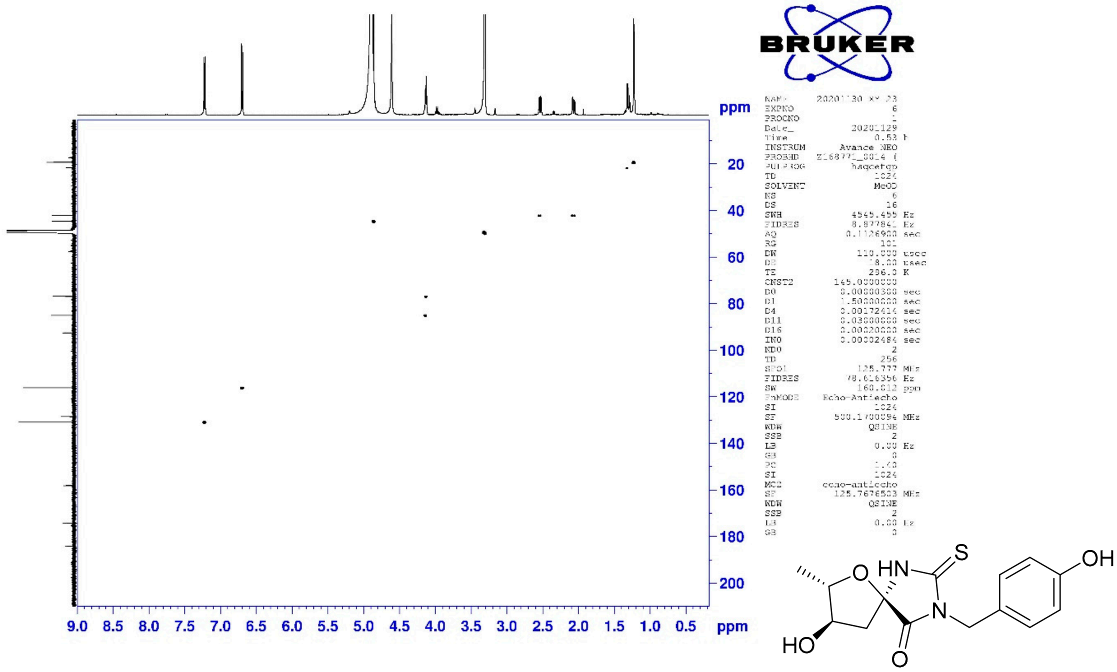

Figure S9. The HSQC spectrum of compound **1** in MeOH-d_4 (500 MHz for ^{1}H).

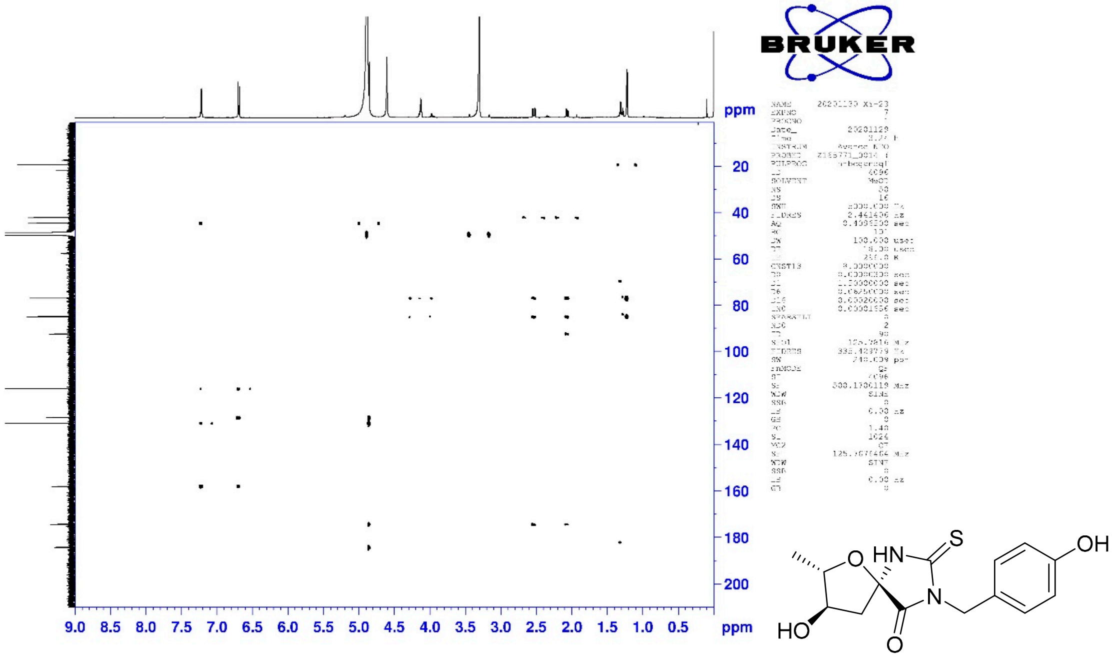

Figure S10. The HMBC spectrum of compound **1** in MeOH-d_4 (500 MHz for ^{1}H).

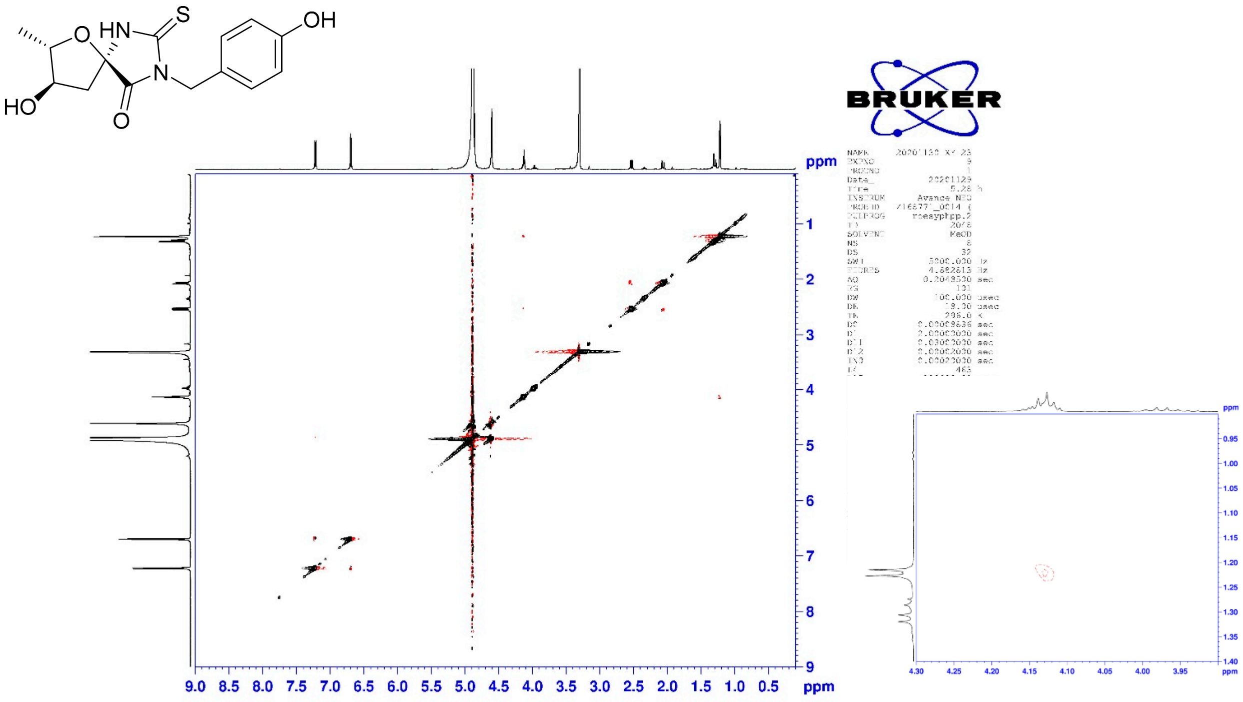

Figure S11. The ROESY spectrum and enlarged ROESY spectrum of compound **1** in MeOH-d_4 (500 MHz).

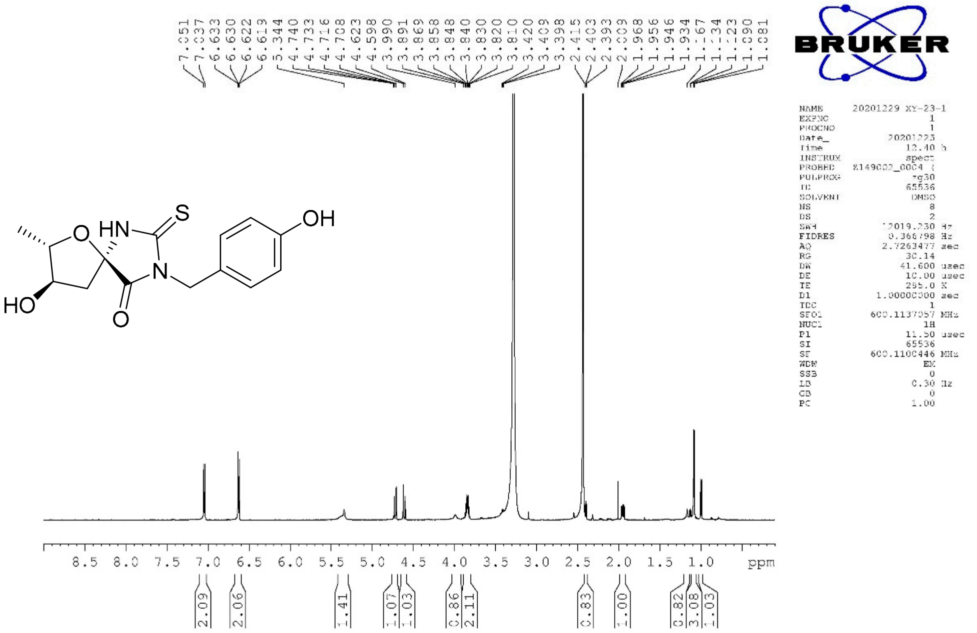

Figure S12. The ^{1}H NMR spectrum of compound **1** in DMSO-d_6 (600 MHz).

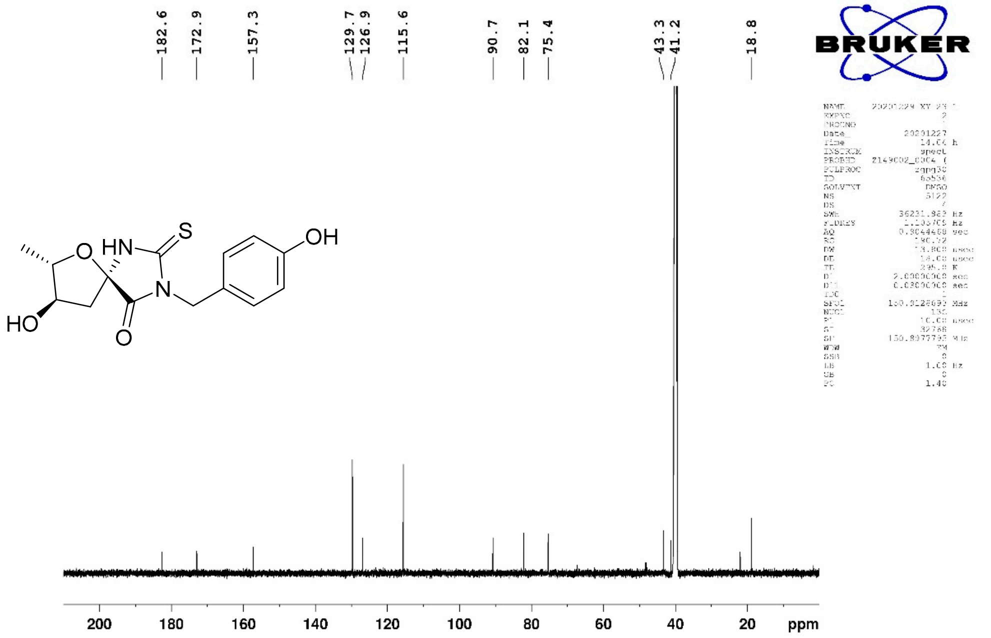

Figure S13. The ^{13}C NMR spectrum of compound **1** in DMSO-d_6 (150 MHz).

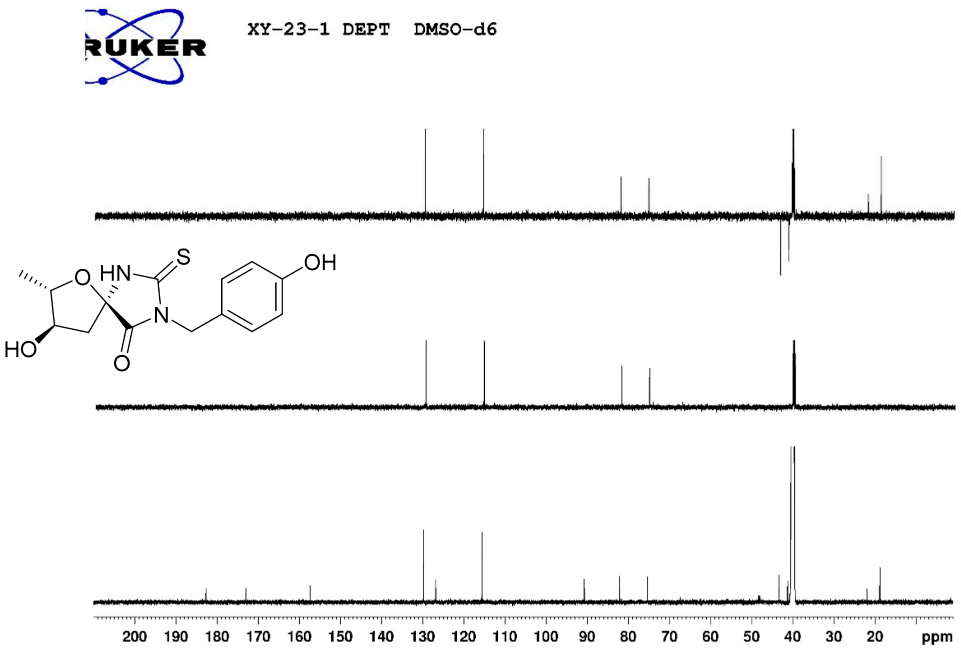

Figure S14. The DEPT spectrum of compound **1** in DMSO-d_6 (150 MHz).

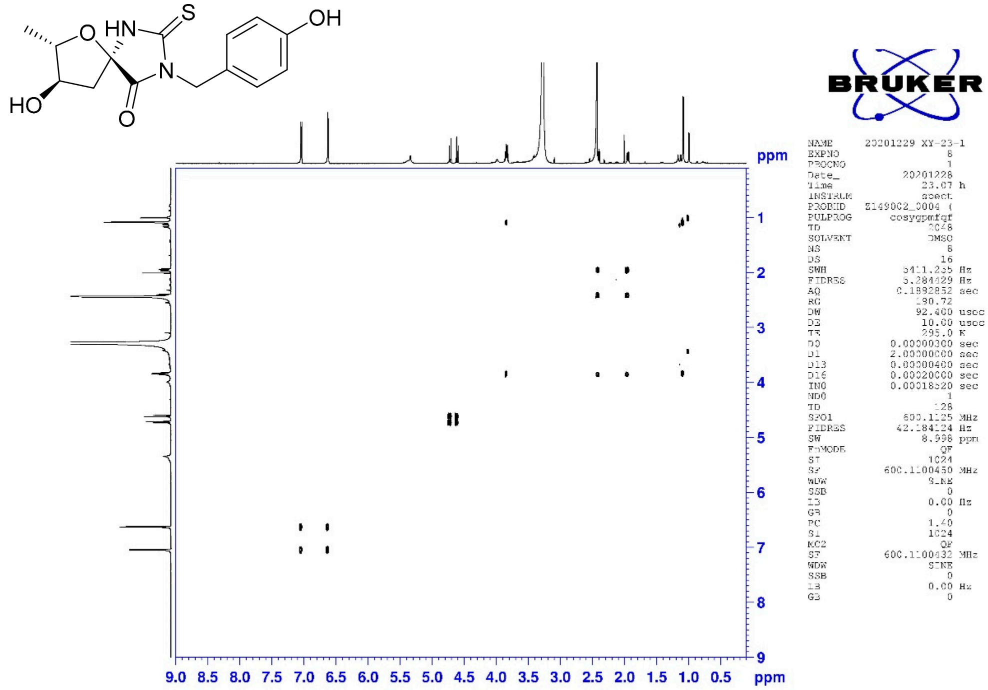

Figure S15. The ^{1}H-^{1}H COSY spectrum of compound **1** in DMSO-d_6 (600 MHz).

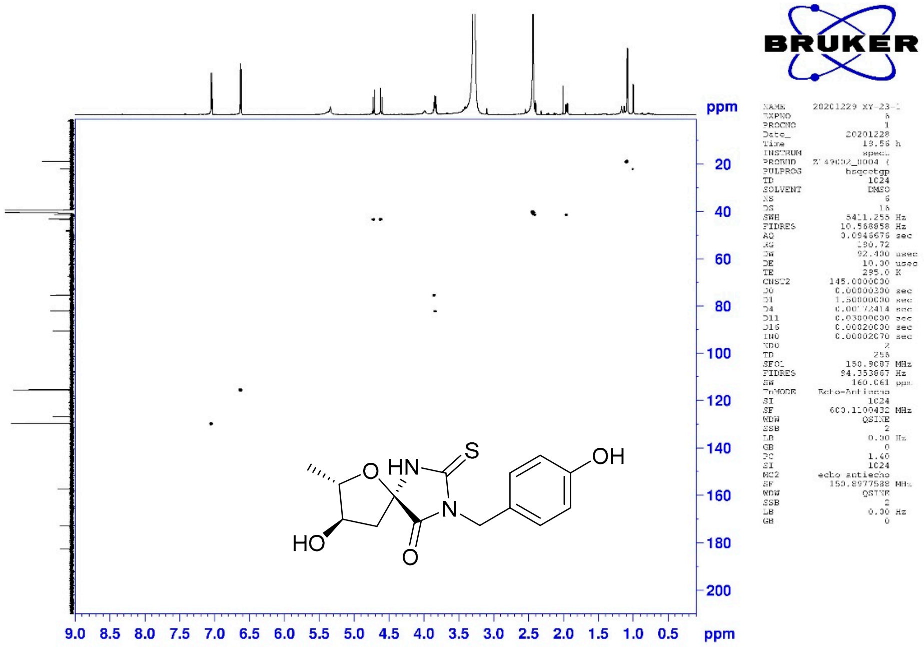

Figure S16. The HSQC spectrum of compound **1** in DMSO-d_6 (600 MHz for ^{1}H).

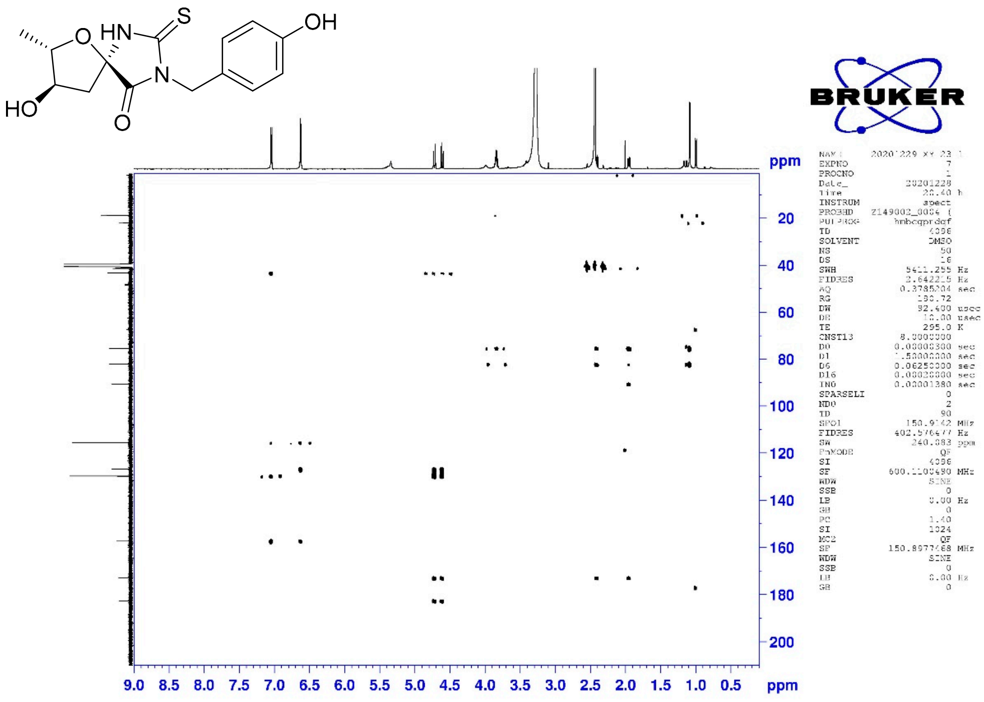

Figure S17. The HMBC spectrum of compound **1** in DMSO-d_6 (600 MHz for ^{1}H).

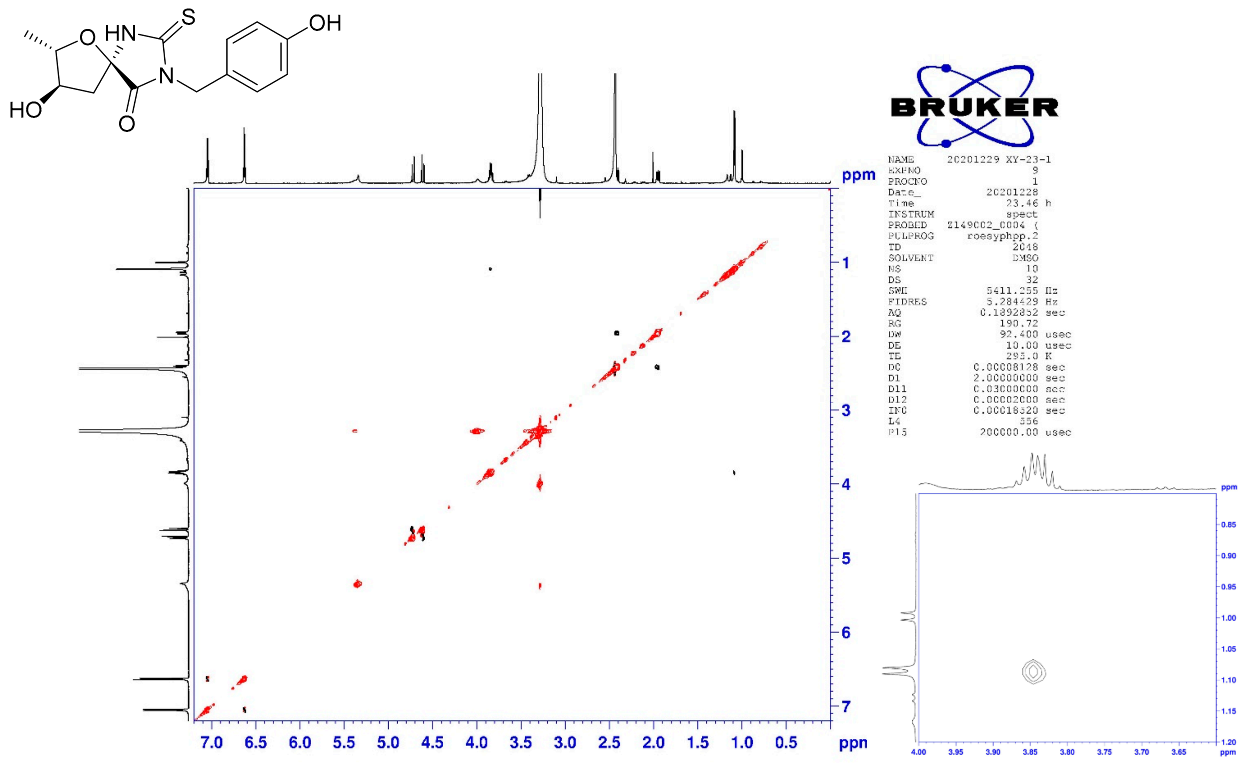

Figure S18. The NOESY spectrum and enlarged NOESY spectrum of compound **1** in DMSO-d_6 (600 MHz).

Qualitative Analysis Report

Chromatograms

Fragmentor Voltage 110 **Collision Energy** 0 **Ionization Mode** ESI

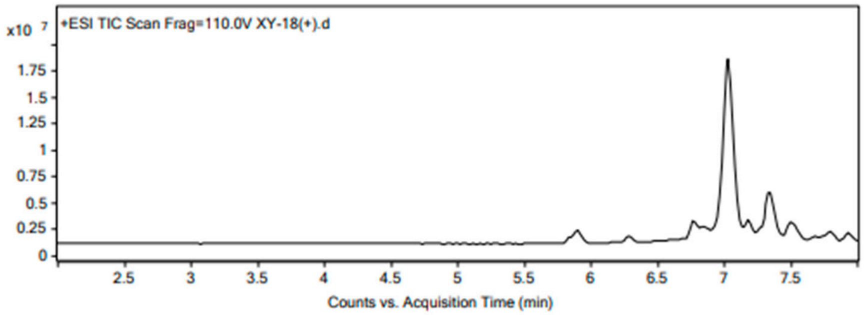

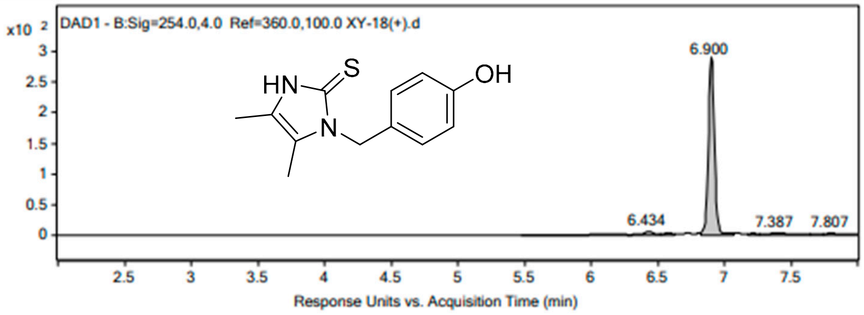

Spectra

Fragmentor Voltage	Collision Energy	Ionization Mode
110	0	ESI

Figure S19. (+)-HRESIMS spectrum of compound **2** (page 1).

Qualitative Analysis Report

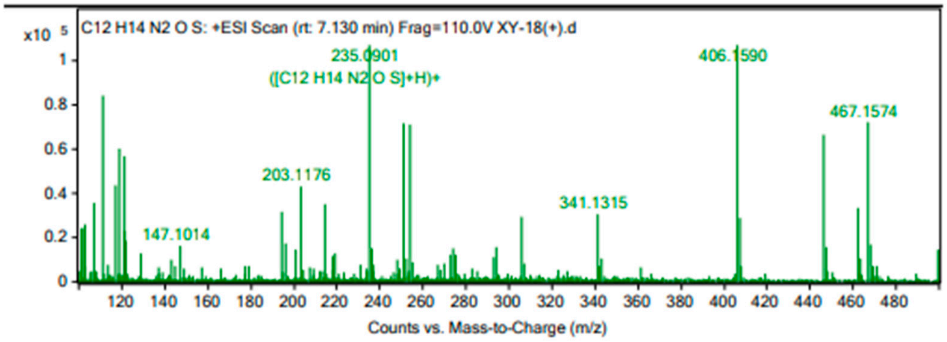

Formula Calculator Element Limits

Element	Min	Max
C	0	60
H	0	120
O	0	10
N	0	5
S	0	5

Formula Calculator Results

Formula	Best	Mass	Tgt Mass	Diff (ppm)	Ion Species	Score
C12 H14 N2 O S	True	234.083	234.0827	-1.17	C12 H15 N2 O S	97.49
C10 H12 N5 S	False	234.0831	234.0813	-7.34	C10 H13 N5 S	85.26
C7 H16 N5 S2	False	234.0832	234.0847	6.58	C7 H17 N5 S2	80.16
C6 H12 N5 O5	False	234.0827	234.0838	4.92	C6 H13 N5 O5	76.2
C9 H16 N O4 S	False	234.0829	234.08	-12.56	C9 H17 N O4 S	70.08
C9 H18 N2 O S2	False	234.0831	234.0861	12.76	C9 H19 N2 O S2	65.82
C8 H14 N2 O6	False	234.0826	234.0852	11.2	C8 H15 N2 O6	61.79
C10 H22 N O S2	True	236.1143	236.1143	0.04	C10 H23 N O S2	47.62
C10 H14 N5 O2	False	236.1143	236.1147	2.03	C10 H15 N5 O2	46.64
C9 H18 N O6	False	236.1143	236.1134	-3.64	C9 H19 N O6	44.55
C8 H20 N4 S2	False	236.1143	236.1129	-5.65	C8 H21 N4 S2	40.56
C12 H16 N2 O3	False	236.1143	236.1161	7.71	C12 H17 N2 O3	35.29

--- End Of Report ---

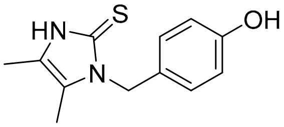

Figure S20. (+)-HRESIMS spectrum of compound **2** (page 2).

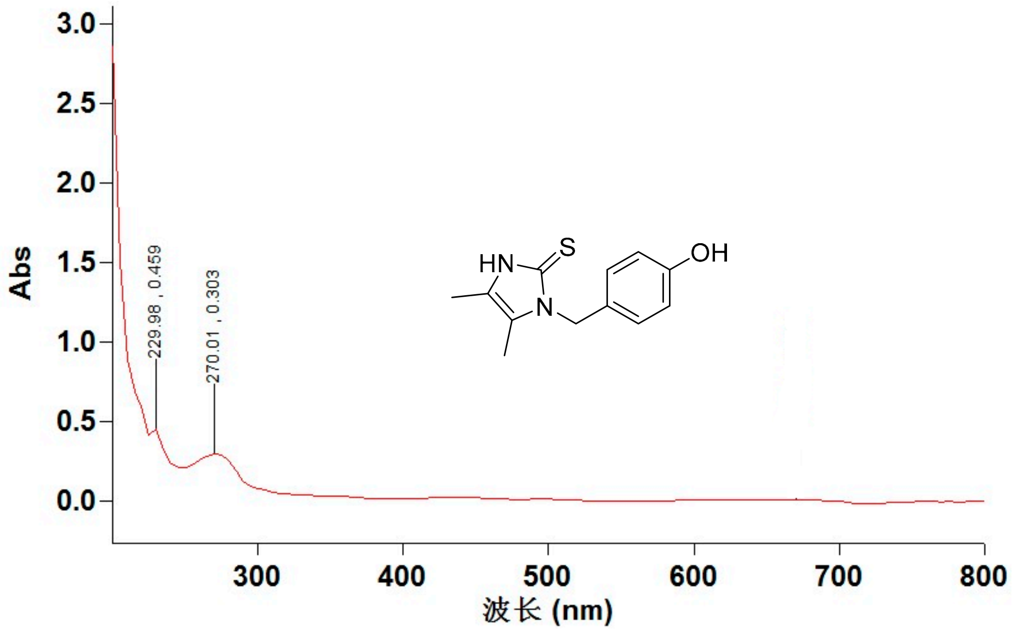

Figure S21. The UV spectrum of compound **2** in MeOH.

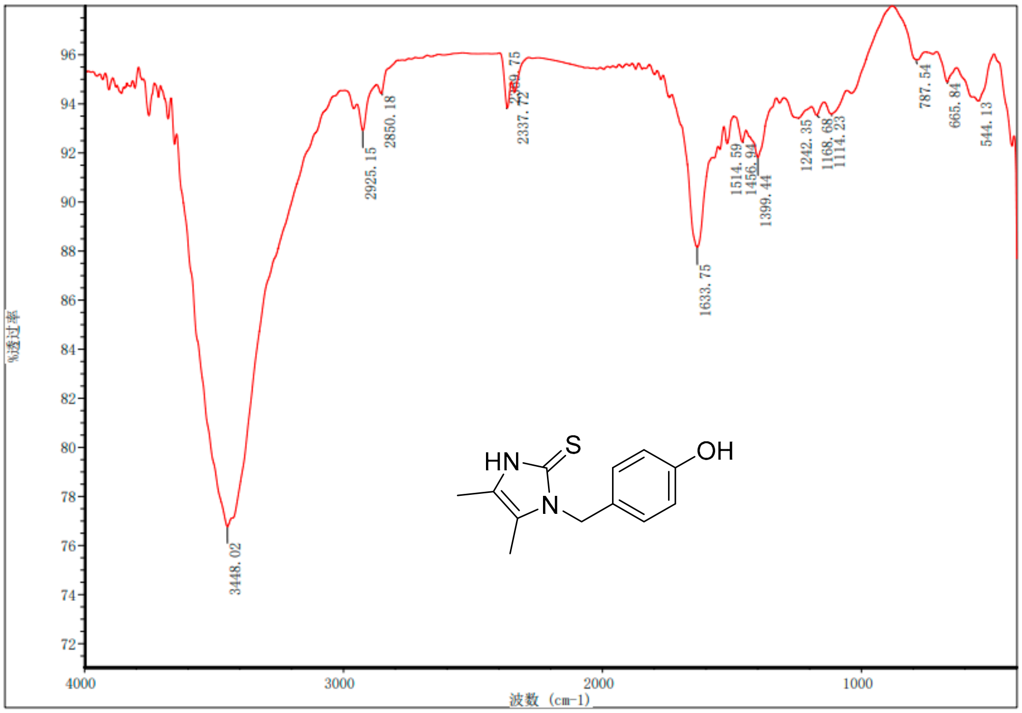

Figure S22. The IR spectrum of compound **2**.

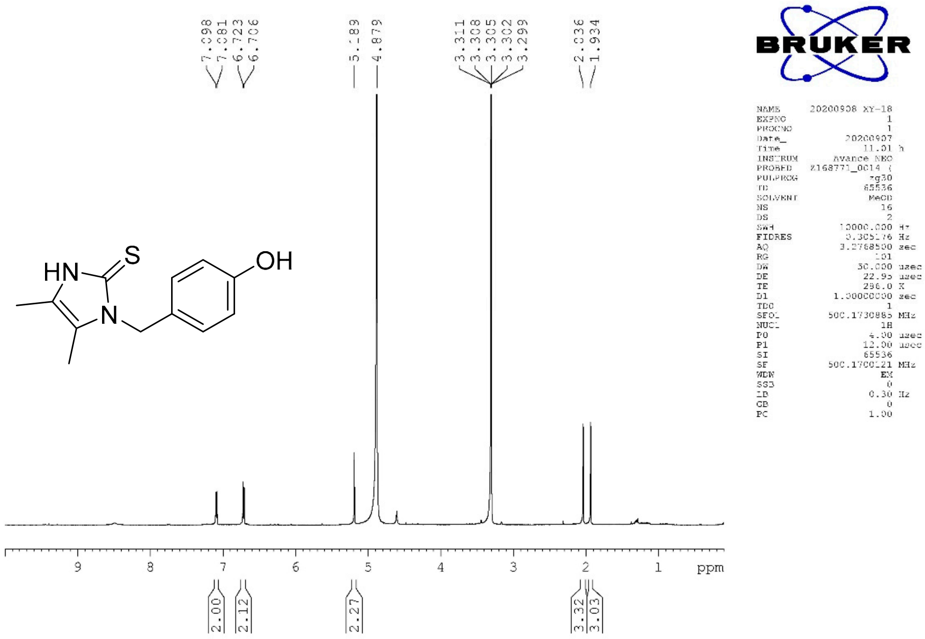

Figure S23. The ^{1}H NMR spectrum of compound **2** in MeOH-d_4 (500 MHz).

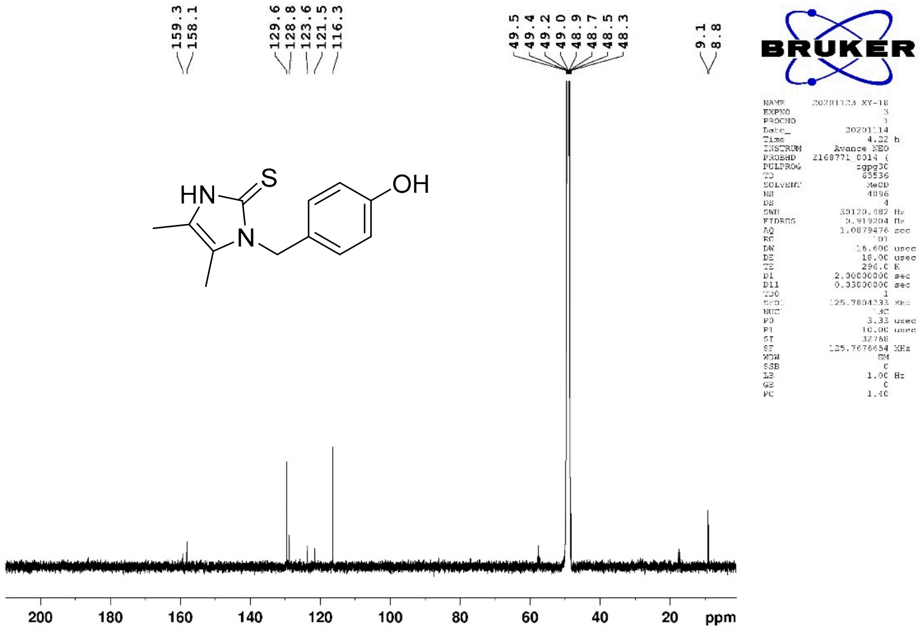

Figure S24. The ^{13}C NMR spectrum of compound **2** in MeOH-d_4 (125 MHz).

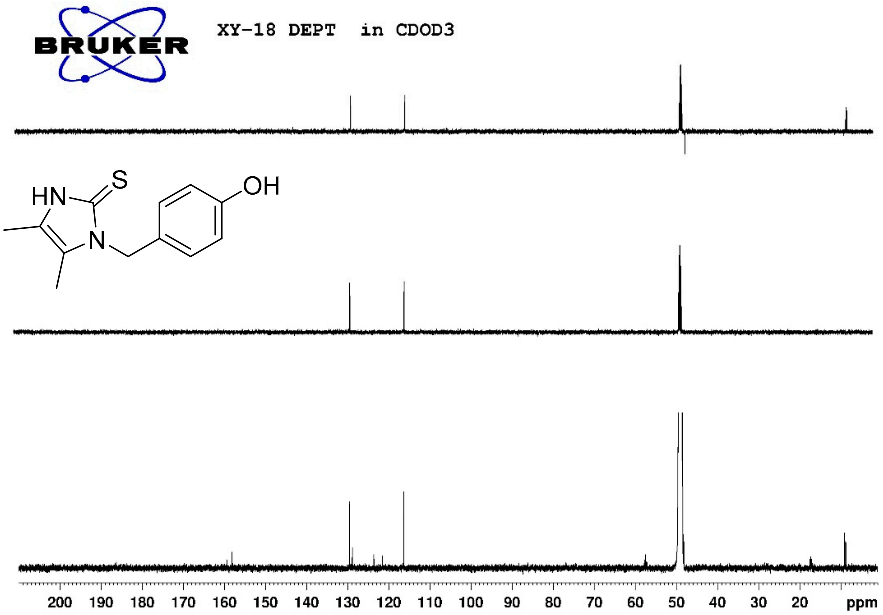

Figure S25. The DEPT spectrum of compound **2** in MeOH-d_4 (125 MHz).

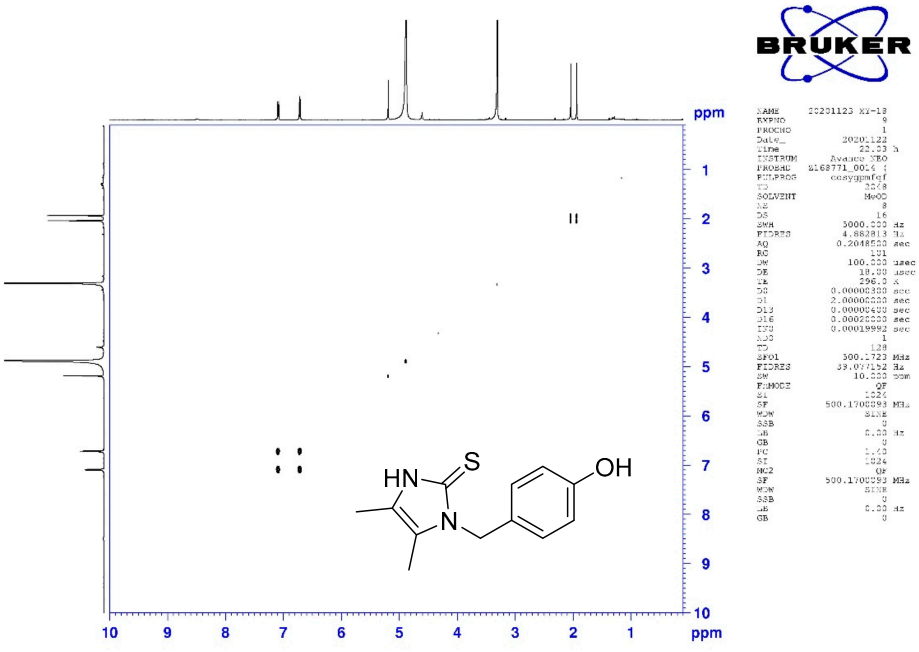

Figure S26. The ^{1}H-^{1}H COSY spectrum of compound **2** in MeOH-d_4 (500 MHz).

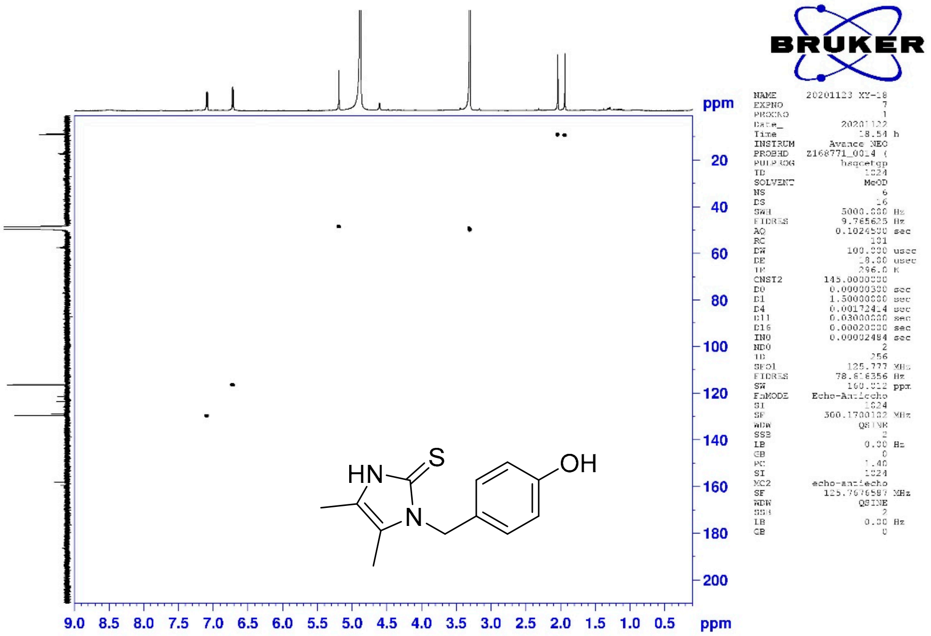

Figure S27. The HSQC spectrum of compound **2** in MeOH-d_4 (500 MHz for ^{1}H).

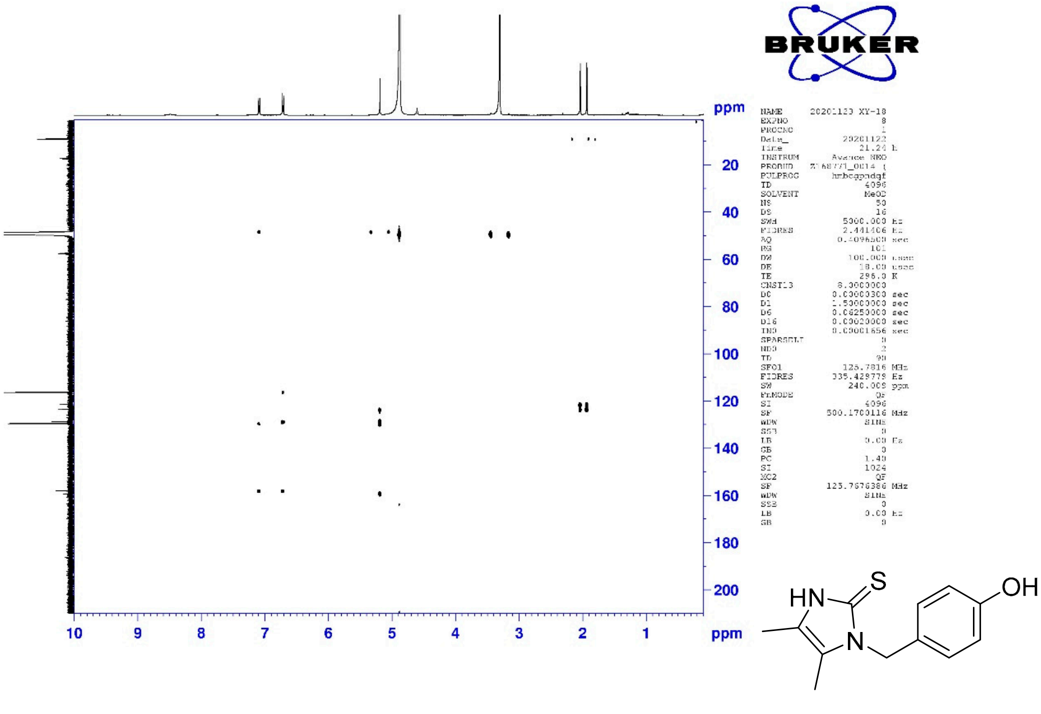

Figure S28. The HMBC spectrum of compound **2** in MeOH-d_4 (500 MHz for ^{1}H).

Table S1. Geometry data of conformers of isomer **1a** and isomer **1b**.

1a-c2 , delta G = 0.0000 kcal/mol, population = 21.81 %

N -1.562738 1.393339 -0.024114
C -1.881717 -0.000459 -0.285641
C -0.612855 -0.442888 -1.034619
N 0.206789 0.659427 -1.112470
C -0.377431 1.777798 -0.514903
O -2.016498 -0.747598 0.903472
C -3.416463 -1.089686 1.137137
C -4.193588 -0.290982 0.083633
C -3.188700 -0.201082 -1.054247
C 1.547383 0.612104 -1.700638
C 2.561277 -0.019893 -0.780158
C 3.156502 -1.237389 -1.105952
C 4.091099 -1.827700 -0.265239
C 4.438431 -1.199513 0.927978
C 3.849613 0.017710 1.270925
C 2.921483 0.598888 0.417631
S 0.288885 3.308855 -0.448185
O -0.379737 -1.547086 -1.467611
C -3.601535 -2.591293 1.043534
O -4.448668 1.052938 0.511585
O 5.361344 -1.820164 1.724742
H -2.234895 2.033134 0.371372
H -3.661176 -0.736790 2.140502
H -5.121121 -0.787769 -0.205518
H -3.140226 -1.142927 -1.599169
H -3.401196 0.608148 -1.747689
H 1.471234 0.047667 -2.628302
H 1.809126 1.641256 -1.938475
H 2.883731 -1.736037 -2.027895
H 4.551925 -2.772772 -0.521377
H 4.119827 0.509105 2.198460
H 2.472881 1.546867 0.687404
H -2.920848 -3.094272 1.730551
H -3.400534 -2.954265 0.034209
H -4.624254 -2.860246 1.313954
H -5.036197 1.029967 1.276555
H 5.520782 -1.293044 2.517850

1a-c10 , delta G = 0.0489 kcal/mol, population = 20.08 %

N -1.595382 1.296291 -0.352961
C -1.981071 -0.015906 0.136261

C -0.770561 -0.351721 1.024010
N 0.094198 0.714196 0.936508
C -0.401299 1.709816 0.092654
O -3.152579 0.030066 0.921093
C -4.272293 -0.574089 0.205248
C -3.745894 -0.807211 -1.215810
C -2.261726 -1.031781 -0.973185
C 1.390852 0.751404 1.623632
C 2.486422 0.082986 0.832177
C 2.679641 -1.298369 0.908682
C 3.673203 -1.924413 0.170158
C 4.497520 -1.167236 -0.660918
C 4.320887 0.212525 -0.745879
C 3.320510 0.826294 -0.001461
S 0.365871 3.146886 -0.276805
O -0.617229 -1.357622 1.677764
C -4.718331 -1.833921 0.920824
O -3.854698 0.369336 -2.027287
O 5.463734 -1.831426 -1.365255
H -2.133867 1.792449 -1.046413
H -5.075082 0.165329 0.198261
H -4.231561 -1.656723 -1.698574
H -2.083552 -2.035664 -0.589859
H -1.654776 -0.869101 -1.859690
H 1.619784 1.798441 1.803568
H 1.243621 0.254143 2.580762
H 2.041125 -1.890541 1.551677
H 3.823069 -2.994406 0.232761
H 4.963093 0.804202 -1.388068
H 3.181833 1.896884 -0.076451
H -5.611130 -2.241120 0.442890
H -4.957998 -1.606925 1.959743
H -3.936469 -2.595100 0.907909
H -4.788825 0.574967 -2.153693
H 5.972679 -1.207115 -1.897674

1a-c5 , delta G = 0.0998 kcal/mol, population = 18.43 %
N -1.570422 1.403864 -0.037794
C -1.891367 0.009821 -0.294642
C -0.616599 -0.440867 -1.028534
N 0.211069 0.656030 -1.096622
C -0.374961 1.779531 -0.510347
O -2.037732 -0.730502 0.897734
C -3.440747 -1.065201 1.123317

C -4.206980 -0.271414 0.058028
C -3.192507 -0.191945 -1.072249
C 1.561423 0.597765 -1.661284
C 2.550538 -0.048636 -0.724197
C 3.134102 -1.271874 -1.040878
C 4.043432 -1.877700 -0.180415
C 4.372523 -1.257637 1.022120
C 3.795786 -0.031666 1.354386
C 2.895600 0.563185 0.483981
S 0.300511 3.306152 -0.436122
O -0.385073 -1.546982 -1.457506
C -3.631006 -2.566710 1.039517
O -4.462045 1.076127 0.474518
O 5.254075 -1.802695 1.915032
H -2.242909 2.047532 0.350716
H -3.691648 -0.703982 2.122195
H -5.133387 -0.767749 -0.235429
H -3.142297 -1.137589 -1.610434
H -3.397125 0.613203 -1.772773
H 1.496838 0.038247 -2.592826
H 1.837796 1.625290 -1.889381
H 2.874127 -1.765705 -1.969009
H 4.489899 -2.830484 -0.440537
H 4.062054 0.442666 2.290007
H 2.454237 1.517008 0.745234
H -4.656557 -2.829812 1.304963
H -2.956987 -3.067165 1.734909
H -3.424567 -2.937857 0.034270
H -5.058365 1.060325 1.232828
H 5.580571 -2.647763 1.580996

1a -c11 , delta G = 0.2102 kcal/mol, population = 15.29 %
N -1.603791 1.263402 0.348397
C -1.953486 -0.004581 -0.269689
C -0.686964 -0.270814 -1.101220
N 0.153578 0.798917 -0.898252
C -0.411361 1.739708 -0.035544
O -2.126553 -1.031675 0.681179
C -3.537716 -1.384810 0.802880
C -4.277934 -0.318899 -0.014240
C -3.252120 0.037996 -1.077956
C 1.499735 0.877785 -1.477446
C 2.525333 0.128660 -0.665340
C 3.204531 0.754526 0.382344

C 4.134449 0.063562 1.145514
C 4.400089 -1.276098 0.866151
C 3.732636 -1.915184 -0.177011
C 2.803728 -1.210832 -0.933360
S 0.276370 3.196664 0.406128
O -0.474077 -1.236324 -1.797543
C -3.754851 -2.805179 0.320121
O -4.509806 0.873393 0.746715
O 5.327498 -1.911473 1.645417
H -2.268902 1.801228 0.883190
H -3.791903 -1.298139 1.860774
H -5.211449 -0.698474 -0.432925
H -3.215749 -0.730300 -1.849185
H -3.433821 1.005462 -1.538244
H 1.427173 0.467457 -2.482984
H 1.744967 1.934617 -1.545329
H 2.997802 1.793525 0.604789
H 4.662849 0.550696 1.954721
H 3.939531 -2.955847 -0.398151
H 2.284156 -1.713324 -1.739101
H -3.540899 -2.899160 -0.745829
H -4.788582 -3.107637 0.497076
H -3.099789 -3.487126 0.862508
H -5.106676 0.666067 1.475894
H 5.430890 -2.826801 1.355797

1a-c9 , delta G = 0.4512 kcal/mol, population = 10.18 %
N -1.596117 1.385555 -0.178536
C -1.897073 0.007552 0.173224
C -0.634040 -0.361058 0.969815
N 0.179517 0.748226 0.959881
C -0.397118 1.802499 0.248972
O -3.032418 -0.097138 1.003256
C -4.158662 -0.656019 0.260950
C -3.682398 -0.700853 -1.196036
C -2.181464 -0.886597 -1.035265
C 1.494346 0.773587 1.606217
C 2.544785 0.044141 0.807266
C 2.986673 0.547727 -0.419091
C 3.945744 -0.122981 -1.162107
C 4.484565 -1.316646 -0.681462
C 4.057130 -1.830442 0.540343
C 3.090311 -1.149119 1.272163
S 0.296981 3.299756 -0.013636

O -0.407026 -1.417853 1.510979
C -4.533078 -2.007574 0.835842
O -3.871442 0.555323 -1.860365
O 5.429088 -1.936081 -1.453042
H -2.180161 1.919491 -0.804086
H -4.985114 0.048371 0.369525
H -4.153563 -1.509721 -1.756680
H -1.947691 -1.918704 -0.777069
H -1.618292 -0.597534 -1.918548
H 1.747859 1.823756 1.734747
H 1.378804 0.319541 2.588906
H 2.574607 1.476528 -0.793566
H 4.289251 0.268257 -2.110907
H 4.473490 -2.757822 0.916784
H 2.754342 -1.559805 2.216204
H -4.727861 -1.917389 1.904578
H -3.732010 -2.734593 0.692466
H -5.436461 -2.383379 0.351888
H -4.817390 0.735344 -1.921936
H 5.731536 -2.743927 -1.019430

1a-c1 , delta G = 0.6081 kcal/mol, population = 7.81 %
N -1.599778 1.402660 -0.141104
C -1.898513 0.013153 0.164531
C -0.651313 -0.369377 0.979354
N 0.153971 0.745273 1.019549
C -0.413854 1.815189 0.325311
O -3.051700 -0.122796 0.964770
C -4.156121 -0.669112 0.181489
C -3.646667 -0.668830 -1.264872
C -2.148333 -0.848093 -1.075132
C 1.453064 0.761705 1.697190
C 2.528122 0.062351 0.903918
C 3.073155 -1.140684 1.349687
C 4.062279 -1.793154 0.625560
C 4.516649 -1.243076 -0.570383
C 3.979057 -0.041712 -1.032898
C 2.994874 0.602316 -0.295158
S 0.275480 3.323717 0.120430
O -0.428762 -1.439546 1.495432
C -4.532028 -2.039417 0.709139
O -3.830628 0.604414 -1.897474
O 5.491058 -1.922018 -1.249363
H -2.173423 1.950216 -0.764488

H -4.990742 0.025421 0.291112
H -4.098555 -1.464784 -1.858874
H -1.912193 -1.885437 -0.841254
H -1.567637 -0.529657 -1.936704
H 1.695281 1.809615 1.860936
H 1.317980 0.279582 2.663883
H 2.716617 -1.578807 2.273690
H 4.484661 -2.726175 0.975131
H 4.333074 0.388856 -1.962490
H 2.584738 1.537428 -0.655741
H -3.721705 -2.755711 0.563823
H -5.420842 -2.408391 0.193972
H -4.751978 -1.981266 1.775205
H -4.776353 0.779649 -1.974464
H 5.724266 -1.445338 -2.055936

1a-c12 , delta G = 1.6893 kcal/mol, population = 1.26 %
N -1.389125 1.568540 -0.274120
C -1.923117 0.244986 -0.048688
C -0.783999 -0.374268 0.786798
N 0.161469 0.609163 0.965986
C -0.207403 1.791448 0.328015
O -3.105373 0.274235 0.742011
C -4.183951 -0.417485 0.066581
C -3.473097 -1.371378 -0.890735
C -2.256957 -0.546184 -1.317350
C 1.418233 0.396845 1.694036
C 2.527832 -0.104281 0.804765
C 3.530068 0.755497 0.360696
C 4.547711 0.301865 -0.470785
C 4.568206 -1.032067 -0.871994
C 3.574192 -1.906292 -0.433357
C 2.567648 -1.441116 0.399411
S 0.665456 3.212227 0.319063
O -0.741673 -1.506114 1.211654
C -5.079282 -1.068772 1.092102
O -4.351673 -1.714804 -1.951463
O 5.538755 -1.541077 -1.690274
H -1.869199 2.289170 -0.789757
H -4.747142 0.308169 -0.531051
H -3.154660 -2.265150 -0.346785
H -1.415779 -1.142021 -1.665077
H -2.551803 0.142864 -2.110043
H 1.683803 1.346344 2.151213

H 1.192303 -0.320846 2.480867
H 3.513419 1.794936 0.661280
H 5.322598 0.981971 -0.805347
H 3.604584 -2.942138 -0.745335
H 1.800164 -2.124910 0.739181
H -5.879749 -1.609003 0.583869
H -5.531730 -0.321614 1.744910
H -4.510939 -1.772816 1.702194
H -3.986673 -2.473120 -2.421613
H 6.167389 -0.847767 -1.927793

1a-c16 , delta G = 1.8179 kcal/mol, population = 1.01 %
N -1.388031 1.546205 -0.334486
C -1.920661 0.231283 -0.061150
C -0.773941 -0.361672 0.782993
N 0.170776 0.628205 0.926482
C -0.203673 1.789785 0.254548
O -3.094440 0.287901 0.741202
C -4.180924 -0.424147 0.100572
C -3.481355 -1.409527 -0.832889
C -2.269424 -0.599949 -1.299551
C 1.429879 0.441124 1.657203
C 2.539675 -0.085015 0.782609
C 2.585613 -1.432980 0.424498
C 3.597127 -1.922240 -0.391930
C 4.588554 -1.058902 -0.857221
C 4.559202 0.288441 -0.504313
C 3.539480 0.764588 0.308185
S 0.666415 3.211006 0.197402
O -0.726467 -1.480388 1.240929
C -5.065312 -1.040708 1.156611
O -4.371832 -1.786588 -1.872040
O 5.613933 -1.480119 -1.658351
H -1.873773 2.250903 -0.866580
H -4.750103 0.282320 -0.514140
H -3.157541 -2.285327 -0.263454
H -1.432748 -1.207693 -1.637370
H -2.572662 0.063544 -2.110666
H 1.694378 1.404899 2.084201
H 1.207262 -0.252194 2.466479
H 1.821362 -2.109142 0.786070
H 3.620983 -2.971976 -0.661423
H 5.335695 0.948872 -0.867764
H 3.516161 1.813623 0.572991

H -5.509772 -0.272202 1.789831
H -4.490699 -1.725216 1.782860
H -5.871878 -1.596218 0.675202
H -4.012527 -2.560204 -2.321177
H 5.532636 -2.427103 -1.827949

1a-c15 , delta G = 1.9089 kcal/mol, population = 0.87 %
N -1.387946 1.537528 -0.260448
C -1.924940 0.216488 -0.026257
C -0.776858 -0.407241 0.794270
N 0.169058 0.576172 0.970540
C -0.204266 1.760252 0.338472
O -3.093116 0.249312 0.785695
C -4.192405 -0.405922 0.106118
C -3.500473 -1.376921 -0.858616
C -2.281994 -0.572074 -1.290140
C 1.429015 0.363734 1.692701
C 2.541717 -0.115245 0.795179
C 3.552620 0.752016 0.387356
C 4.575012 0.318998 -0.449374
C 4.591100 -1.001402 -0.892980
C 3.587974 -1.883031 -0.491304
C 2.577079 -1.438659 0.347455
S 0.666268 3.182081 0.331952
O -0.730295 -1.540987 1.213116
C -5.104832 -1.032776 1.131745
O -4.286575 -1.720060 -1.990360
O 5.565728 -1.490001 -1.718779
H -1.875529 2.262539 -0.762779
H -4.733462 0.337716 -0.489656
H -3.191569 -2.274279 -0.314456
H -1.454728 -1.182186 -1.644907
H -2.572226 0.119383 -2.082794
H 1.688327 1.308306 2.163551
H 1.209665 -0.367098 2.469285
H 3.539250 1.781582 0.720330
H 5.356487 1.004851 -0.755683
H 3.614808 -2.908451 -0.836286
H 1.803244 -2.128767 0.658985
H -4.563044 -1.770517 1.726088
H -5.938658 -1.528420 0.630316
H -5.517308 -0.276050 1.799779
H -4.903587 -2.417372 -1.739610
H 6.200028 -0.793106 -1.929198

1a-c13 ,   delta G =   1.9421   kcal/mol, population =   0.82 %

N -1.459361 1.374538 0.869365
C -1.946188 0.326518 0.001833
C -0.764289 0.210583 -0.982422
N 0.181690 1.126205 -0.582371
C -0.241190 1.837964 0.537650
O -2.092640 -0.909405 0.688444
C -3.437073 -1.423095 0.528016
C -3.934956 -0.766453 -0.757628
C -3.287417 0.618044 -0.680322
C 1.492465 1.264738 -1.229414
C 2.522654 0.322530 -0.660675
C 3.433033 0.755811 0.301475
C 4.371180 -0.114943 0.843416
C 4.404299 -1.443121 0.423159
C 3.502800 -1.891061 -0.541587
C 2.574294 -1.010498 -1.076201
S 0.602768 3.058456 1.298859
O -0.685863 -0.548346 -1.920966
C -3.398143 -2.931732 0.518168
O -5.354373 -0.751887 -0.759735
O 5.300373 -2.350281 0.917784
H -1.990393 1.768350 1.629945
H -4.053594 -1.059958 1.358200
H -3.552098 -1.318192 -1.620817
H -3.162262 1.103493 -1.645880
H -3.891361 1.260924 -0.038691
H 1.329088 1.072266 -2.288330
H 1.796977 2.300521 -1.104062
H 3.405210 1.784110 0.637672
H 5.075412 0.236640 1.588784
H 3.542292 -2.923072 -0.865019
H 1.876092 -1.362784 -1.824757
H -2.767337 -3.292336 -0.296059
H -4.408670 -3.319733 0.379445
H -3.010453 -3.317062 1.461747
H -5.659591 -0.536154 -1.648415
H 5.865899 -1.929386 1.577444

1a-c18 ,   delta G =   2.0350   kcal/mol, population =   0.70 %

N -1.460522 1.376139 0.822021
C -1.941406 0.302164 -0.016999
C -0.760880 0.170443 -1.001610

N 0.180645 1.100336 -0.624895
C -0.244738 1.837036 0.477977
O -2.073468 -0.918990 0.698366
C -3.425254 -1.426601 0.578909
C -3.934358 -0.810371 -0.730572
C -3.286984 0.567616 -0.700993
C 1.490496 1.229346 -1.275836
C 2.526845 0.310633 -0.680398
C 3.440075 0.779494 0.262107
C 4.383907 -0.069057 0.828881
C 4.419829 -1.410634 0.453972
C 3.515519 -1.894249 -0.490742
C 2.581453 -1.035592 -1.050665
S 0.594127 3.079309 1.208483
O -0.679824 -0.610424 -1.921539
C -3.393039 -2.934623 0.629975
O -5.347476 -0.683994 -0.794839
O 5.321340 -2.297377 0.975049
H -1.991730 1.784591 1.574720
H -4.027586 -1.024854 1.401488
H -3.558118 -1.391548 -1.577455
H -3.170549 1.019458 -1.683270
H -3.888738 1.232125 -0.079174
H 1.328230 1.005658 -2.328770
H 1.788252 2.270246 -1.180257
H 3.410066 1.818482 0.563408
H 5.090240 0.310104 1.558576
H 3.557286 -2.936513 -0.779107
H 1.881377 -1.415574 -1.783780
H -2.976565 -3.282669 1.575739
H -2.790532 -3.332636 -0.188373
H -4.407575 -3.329166 0.544308
H -5.723960 -1.529761 -1.064275
H 5.888740 -1.852168 1.616899

1a-c14 , delta G = 2.0563 kcal/mol, population = 0.68 %
N -1.461410 1.379693 0.879500
C -1.942805 0.333792 0.006277
C -0.753100 0.218035 -0.968657
N 0.188704 1.135073 -0.562365
C -0.242116 1.845484 0.555490
O -2.097790 -0.902773 0.689471
C -3.441739 -1.413762 0.516115
C -3.926938 -0.753675 -0.772571

C -3.277752 0.629520 -0.686696
C 1.503848 1.272622 -1.201061
C 2.522070 0.311118 -0.643227
C 3.385582 0.699491 0.382608
C 4.308685 -0.188637 0.915942
C 4.378956 -1.490613 0.423932
C 3.527227 -1.893650 -0.603598
C 2.608932 -0.993471 -1.129327
S 0.594701 3.066889 1.323337
O -0.666611 -0.540741 -1.906602
C -3.405315 -2.922416 0.503107
O -5.346227 -0.736566 -0.787930
O 5.304518 -2.327616 0.983668
H -1.998202 1.773269 1.636134
H -4.064891 -1.051322 1.341603
H -3.537094 -1.304305 -1.633372
H -3.143358 1.116825 -1.650071
H -3.886040 1.272210 -0.048878
H 1.345159 1.099484 -2.263904
H 1.815940 2.303661 -1.056276
H 3.328267 1.707305 0.772899
H 4.978762 0.114719 1.709818
H 3.585554 -2.903684 -0.992794
H 1.947900 -1.312207 -1.925114
H -2.767962 -3.282154 -0.306407
H -4.415208 -3.308401 0.354431
H -3.026693 -3.310713 1.449148
H -5.642497 -0.516591 -1.678627
H 5.256238 -3.197966 0.568631

1a-c19 , delta G = 2.1593 kcal/mol, population = 0.57 %
N -1.386186 1.518993 -0.308418
C -1.921887 0.204420 -0.037887
C -0.768095 -0.399440 0.789451
N 0.177125 0.588998 0.938559
C -0.200535 1.757535 0.280576
O -3.083615 0.258221 0.782510
C -4.189384 -0.409888 0.126560
C -3.506407 -1.406231 -0.818455
C -2.289730 -0.614742 -1.278923
C 1.439232 0.395300 1.662124
C 2.550891 -0.105010 0.774863
C 3.557985 0.755612 0.337634
C 4.580402 0.303692 -0.485332

C 4.604758 -1.030005 -0.886811
C 3.606069 -1.904281 -0.458847
C 2.592096 -1.439463 0.368657
S 0.667732 3.180013 0.237378
O -0.717482 -1.523430 1.233484
C -5.096743 -1.008392 1.173370
O -4.301180 -1.776412 -1.935487
O 5.632516 -1.427572 -1.696919
H -1.877918 2.232095 -0.823589
H -4.732228 0.320537 -0.483789
H -3.195069 -2.290117 -0.253953
H -1.466199 -1.235136 -1.624410
H -2.584624 0.057211 -2.086492
H 1.698889 1.351474 2.108790
H 1.222457 -0.316099 2.457210
H 3.538308 1.794643 0.639562
H 5.362300 0.972769 -0.820238
H 3.626381 -2.943751 -0.765798
H 1.822693 -2.124583 0.701380
H -5.501885 -0.234145 1.825635
H -4.553363 -1.733408 1.781765
H -5.935825 -1.512997 0.689974
H -4.918719 -2.464790 -1.662320
H 5.545680 -2.366740 -1.903215

1a-c20 , delta G = 2.2283 kcal/mol, population = 0.51 %
N -1.448304 1.513381 0.564351
C -1.934393 0.297315 -0.047084
C -0.758721 -0.025893 -0.992510
N 0.182821 0.961067 -0.812974
C -0.236542 1.899526 0.127013
O -2.065624 -0.760585 0.892798
C -3.419809 -1.275674 0.882185
C -3.934339 -0.925943 -0.520096
C -3.282683 0.428453 -0.764521
C 1.488424 0.957993 -1.485285
C 2.522663 0.155362 -0.737844
C 2.604042 -1.227596 -0.901708
C 3.536855 -1.978236 -0.197243
C 4.409168 -1.343634 0.686039
C 4.344945 0.038097 0.855382
C 3.406975 0.775235 0.146648
S 0.603587 3.262454 0.593413
O -0.681108 -0.972765 -1.740841

C -3.392654 -2.744636 1.227252
O -5.347337 -0.808879 -0.601087
O 5.349618 -2.024933 1.408507
H -1.976095 2.062440 1.224301
H -4.015993 -0.717867 1.613092
H -3.564446 -1.663374 -1.238413
H -3.169468 0.677945 -1.816906
H -3.879495 1.204703 -0.283188
H 1.317394 0.546290 -2.478287
H 1.792928 1.996703 -1.583872
H 1.927488 -1.725500 -1.584519
H 3.590591 -3.051921 -0.336332
H 5.030957 0.520633 1.539348
H 3.354371 1.846991 0.287396
H -2.972599 -2.902967 2.220999
H -2.795930 -3.297784 0.499864
H -4.409053 -3.143693 1.224732
H -5.728053 -1.689593 -0.696293
H 5.294492 -2.969152 1.214860

1b-c5 , delta G = 0.0000 kcal/mol, population = 31.84 %
N -1.501573 1.513260 0.676728
C -2.003601 0.241106 0.214456
C -0.868459 -0.167046 -0.744167
N 0.036762 0.856091 -0.764785
C -0.347482 1.888932 0.097281
O -3.202178 0.383148 -0.530488
C -4.204983 -0.580451 -0.087955
C -3.414384 -1.635944 0.698510
C -2.288406 -0.797759 1.306678
C 1.280286 0.818604 -1.548056
C 2.387285 0.085715 -0.834531
C 2.539233 -1.292486 -0.984140
C 3.540955 -1.979671 -0.309787
C 4.411787 -1.284710 0.527825
C 4.275973 0.093839 0.683754
C 3.270627 0.767243 0.005248
S 0.483147 3.309380 0.348695
O -0.812803 -1.204920 -1.375347
C -5.284527 0.120640 0.715171
O -2.938006 -2.694432 -0.124872
O 5.419030 -1.901374 1.216824
H -1.996430 2.111401 1.319679
H -4.622308 -1.025757 -0.990614

H -4.032467 -2.101775 1.463552
H -2.631172 -0.282046 2.203077
H -1.413382 -1.390349 1.564240
H 1.034091 0.332671 -2.490199
H 1.552308 1.852017 -1.745896
H 1.863723 -1.838054 -1.630547
H 3.647977 -3.050742 -0.436510
H 4.962016 0.623974 1.331457
H 3.165449 1.836861 0.132556
H -5.709618 0.940590 0.135939
H -4.888129 0.527722 1.647340
H -6.085004 -0.581084 0.956779
H -2.248764 -2.343058 -0.716640
H 5.417256 -2.848258 1.027693

1b-c1 , delta G = 0.1525 kcal/mol, population = 24.61 %
N -1.494548 1.553039 0.619806
C -2.007367 0.263123 0.223401
C -0.889992 -0.190794 -0.735578
N 0.016038 0.828608 -0.818855
C -0.351340 1.900152 0.002230
O -3.218256 0.376037 -0.506559
C -4.215140 -0.565254 -0.006297
C -3.414026 -1.586136 0.814532
C -2.276165 -0.724874 1.365534
C 1.244634 0.754403 -1.622746
C 2.368146 0.062804 -0.893968
C 3.269397 0.790480 -0.117776
C 4.291843 0.155082 0.575855
C 4.423963 -1.230251 0.496711
C 3.533834 -1.971310 -0.278873
C 2.517596 -1.323924 -0.966527
S 0.484806 3.329331 0.173243
O -0.846971 -1.256351 -1.319749
C -5.281699 0.172959 0.780741
O -2.953911 -2.682757 0.032850
O 5.409052 -1.913797 1.153953
H -1.977726 2.181319 1.242587
H -4.646676 -1.049768 -0.881688
H -4.020631 -2.014686 1.609949
H -2.602702 -0.168363 2.243406
H -1.398307 -1.307949 1.634866
H 0.982108 0.219807 -2.533619
H 1.509021 1.776943 -1.878818

H 3.167653 1.865720 -0.049230
H 4.987000 0.733240 1.173574
H 3.649775 -3.045645 -0.338208
H 1.826956 -1.904248 -1.565002
H -6.079460 -0.515784 1.064912
H -5.714184 0.967220 0.171946
H -4.871184 0.620254 1.688036
H -2.274028 -2.361038 -0.585942
H 5.963545 -1.297911 1.649508

1b-c2 , delta G = 0.1845 kcal/mol, population = 23.31 %
N -1.567580 1.668398 -0.106005
C -2.038359 0.318034 0.086751
C -0.873946 -0.260937 0.914199
N 0.062708 0.724973 1.046512
C -0.356874 1.907652 0.428186
O -2.122310 -0.391138 -1.139026
C -3.393943 -1.100111 -1.242803
C -3.943837 -1.132540 0.190419
C -3.407012 0.179930 0.765424
C 1.367017 0.521283 1.693421
C 2.393154 -0.050130 0.748783
C 3.241710 0.786525 0.025282
C 4.169321 0.265123 -0.868206
C 4.256484 -1.114379 -1.047289
C 3.418594 -1.964457 -0.327236
C 2.497840 -1.430441 0.562332
S 0.485024 3.343323 0.397333
O -0.807084 -1.395286 1.347309
C -4.295100 -0.407666 -2.247703
O -3.512477 -2.276100 0.919053
O 5.147015 -1.689467 -1.910903
H -2.082664 2.378006 -0.603105
H -3.156400 -2.111833 -1.570420
H -5.031646 -1.162294 0.195096
H -4.035701 1.018550 0.467540
H -3.341142 0.168208 1.851057
H 1.676929 1.489721 2.077277
H 1.190377 -0.149678 2.531927
H 3.172263 1.858564 0.155731
H 4.824705 0.926948 -1.422563
H 3.499688 -3.034179 -0.469584
H 1.846712 -2.094305 1.116660
H -5.203934 -0.992826 -2.400040

H -3.783568 -0.312335 -3.205762
H -4.578196 0.590677 -1.909017
H -2.553975 -2.201090 1.075967
H 5.668044 -1.006132 -2.351241

1b-c6 , delta G = 0.2679 kcal/mol, population = 20.24 %
N -1.573284 1.626617 -0.365574
C -2.039538 0.325405 0.049094
C -0.860904 -0.115402 0.939080
N 0.076011 0.878125 0.897540
C -0.355116 1.947390 0.105136
O -2.144011 -0.571376 -1.045085
C -3.418038 -1.283023 -1.013293
C -3.942751 -1.084818 0.415958
C -3.396300 0.301204 0.764416
C 1.389700 0.779249 1.549826
C 2.401632 0.057348 0.697361
C 2.512233 -1.331973 0.749527
C 3.422958 -2.008015 -0.052823
C 4.242901 -1.290701 -0.922419
C 4.147426 0.098601 -0.982499
C 3.232705 0.760794 -0.176573
S 0.483369 3.359333 -0.166964
O -0.785314 -1.166118 1.546598
C -4.335810 -0.755960 -2.100180
O -3.497989 -2.098421 1.310255
O 5.160900 -1.895968 -1.735118
H -2.097905 2.248904 -0.960116
H -3.187118 -2.334831 -1.179727
H -5.030296 -1.110898 0.444339
H -4.030141 1.083075 0.347068
H -3.311676 0.463050 1.836669
H 1.705735 1.796718 1.764791
H 1.225428 0.255676 2.489693
H 1.875721 -1.894270 1.420756
H 3.499378 -3.087909 -0.000826
H 4.792970 0.645381 -1.657409
H 3.157222 1.839054 -0.230265
H -5.248131 -1.353927 -2.141402
H -3.841168 -0.817046 -3.069823
H -4.611270 0.284882 -1.920544
H -2.536993 -2.001377 1.436874
H 5.138234 -2.852284 -1.603845
